# Engaging Gut‐to‐Brain Signalling to Treat Alcohol Use Disorder

**DOI:** 10.1111/adb.70144

**Published:** 2026-03-25

**Authors:** Paula L. Hoffman, Giordano de Guglielmo, Valentina Vengeliene, Wolfgang Kunze, Christina L. Lebonville, Jerome D. Swinny, Amanda J. Roberts, Olivier George, Karen‐Anne McVey Neufeld, Alexandra Dunbar, Laura M. Saba, Ruolin Ma, Leandro F. Vendruscolo, Howard C. Becker, Rainer Spanagel, Boris Tabakoff

**Affiliations:** ^1^ Lohocla Research Corporation Aurora Colorado USA; ^2^ Department of Pharmacology University of Colorado‐Anschutz Medical Campus Aurora Colorado USA; ^3^ Department of Psychiatry, School of Medicine University of California San Diego La Jolla California USA; ^4^ Institute for Psychopharmacology, Central Institute of Mental Health, Medical Faculty Mannheim Mannheim Germany; ^5^ The Brain‐Body Institute, Gut, Brain & Aging Laboratory McMaster University Hamilton Ontario Canada; ^6^ Department of Psychiatry & Behavioral Sciences Medical University of South Carolina Charleston South Carolina USA; ^7^ Ralph H. Johnson Department of Veterans Affairs Medical Center Charleston South Carolina USA; ^8^ School of Medicine, Pharmacy & Biomedical Sciences University of Portsmouth Portsmouth UK; ^9^ Animal Models Core Facility, Scripps Research La Jolla California USA; ^10^ Department of Psychiatry University of California San Diego La Jolla California USA; ^11^ Department of Pharmaceutical Sciences, Skaggs School of Pharmacy and Pharmaceutical Sciences University of Colorado Anschutz Medical Campus Aurora Colorado USA; ^12^ Stress and Addiction Neuroscience Unit, National Institute on Drug Abuse Intramural Research Program NIH Baltimore Maryland USA; ^13^ National Institute on Alcohol Abuse and Alcoholism Division of Intramural Clinical and Biological Research NIH Bethesda Maryland USA; ^14^ German Center for Mental Health (DZPG), Partner Site Mannheim, Heidelberg, Ulm Berlin Germany; ^15^ Institute for Behavioral Genetics University of Colorado‐Boulder Boulder Colorado USA

**Keywords:** AUD, gut–brain signalling, hippocampal inflammatory mediators, Nezavist, NTS activation, peripheral inflammation, vagal activation

## Abstract

According to the 2023 National Survey on Drug Use and Health (NSDUH), 28.9 million people ages 12 and older in the United States had alcohol use disorder (AUD) in the past year. Although chronic alcohol use contributes to numerous health disorders as well as being an economic burden, there are few medications approved for treatment of AUD, and these medications are not uniformly effective and are not widely used. We now describe studies of a small molecule, novel chemical entity called Nezavist, which shows promise as a medication to treat AUD and possibly other addictive disorders. Nezavist acts as a positive allosteric modulator at a novel site on the GABA_A_ receptor, but pharmacokinetic analysis demonstrates that Nezavist does not enter the CNS. However, Nezavist effectively reduces relapse to chronic alcohol consumption in alcohol‐dependent animals in two widely used models. An important goal of the current studies is to provide evidence for the hypothesis that Nezavist acts in the intestine to stimulate vagus nerve afferents that project to the brainstem (nucleus tractus solitarius), leading to reduced inflammation in the brain that may alleviate alcohol ‘craving’ during abstinence from alcohol. It is hoped that the presentation of the current results will stimulate interest in further confirmation of the mechanism of action of Nezavist, with the intent of developing a new and effective medication for treatment for AUD.

## 1 Introduction

We have previously reported on the synthesis [1] and molecular pharmacology of a new chemical entity (2‐ethylcarboxylate‐5,7‐dichloro‐4‐([{diphenylamino}carbonyl]amino)quinoline) which we refer to as Nezavist (acronym = DCUK‐OEt). Nezavist was found to have selective actions as a positive allosteric modulator (PAM) at the GABA_A_ receptor [2]. The actions of Nezavist were traced to a site on the receptor involving the interface of the α and *β* subunits, which was differentiated from the benzodiazepine binding site [2]. Nezavist selectively potentiated effects of GABA on GABA_A_ receptors containing particular subunit combinations and functioned in the presence of either *γ* or *δ* subunits [2]. This profile of Nezavist's in vitro actions generated a hypothesis that Nezavist may be efficacious in treating certain sequelae of alcohol use disorder (AUD). Benzodiazepines are routinely used to treat the hyperexcitability and anxiety associated with the alcohol withdrawal syndrome [3, 4, 5] and our early studies with alcohol‐dependent mice showed that a Nezavist analogue, at high dose levels, was also efficacious in allaying withdrawal hyperexcitability [1].

Recent work has posited that the negative affective state of alcohol withdrawal, also termed hyperkatifeia [6] can drive alcohol relapse/escalation of alcohol consumption by humans and other animals [7]. We now report on the effect of Nezavist in two well‐established models of abstinence‐induced escalation of alcohol intake by alcohol‐dependent rats. We also performed a series of additional behavioural assessments of Nezavist in mice and rats, as well as studies on Nezavist pharmacokinetics, metabolism and tissue distribution. The latter work provided a surprising result, i.e., that Nezavist and its major metabolite are excluded from brain and, in fact, little Nezavist is found in the circulation of animals treated with Nezavist by the oral or intraperitoneal routes at doses producing behavioural effects. We therefore considered systems functioning outside the CNS that can affect an animal's behaviour.

There has been a plethora of research regarding communication between gut and brain which can impact alcohol consumption [8, 9, 10]. Communication pathways have been ascribed to mediators of inflammation and neuroimmune signalling [11, 12], hormones that influence appetite (e.g., CCK [13, 14], GLP‐1 [15, 16, 17, 18, 19], ghrelin [18, 20, 21], leptin [22, 23]) and afferent vagal communication between gut and brain [24, 25]. Our attention became focused on the vagal link between gut and brain since GABA_A_ receptors are part of the enteric nervous system that controls gut motility, sensation and secretion, as well as the microbiota–gut–brain axis [26, 27], and the enteric nervous system acts in concert with vagal sensory and motor function to link the brain and the gut [27, 28]. In addition, GABA_A_ receptors control hormone (CCK) release from enteroendocrine cells [29] that then activates sensory neurons of the enteric nervous system and vagal afferents [30]. Finally, there is some evidence that vagal afferent neurons transcribe GABA_A_ receptor subunit RNA and may themselves express GABA_A_ receptors [31]. In the present work, we present data on gut motility, electrophysiological responses of certain vagal afferent fibres to Nezavist, brainstem c‐Fos responses to Nezavist in anatomical areas receiving afferent vagal input and responses to Nezavist by the CNS and peripheral immune systems, to draw attention to a possible peripheral mechanism of hyperkatifeia in driving relapse to alcohol drinking and high levels of alcohol consumption.

## 2 Materials and Methods

### 2.1 Nezavist (DCUK‐OEt) Synthesis

Synthesis of Nezavist is described in US Patent # 6962930.

### 2.2 Abstinence‐Induced Escalation of Alcohol Intake: Rat Studies

#### 2.2.1 Alcohol Deprivation Effect (ADE) (Laboratory of Prof. Dr. Rainer Spanagel, Central Institute of Mental Health, Mannheim, Germany)

##### 2.2.1.1 Animals

Methods for the measurement of the alcohol (ethanol) deprivation effect are described in detail in Holter et al. [32] and Spanagel and Holter [33]. In brief, 2‐month‐old male Wistar rats (from the breeding colony at the Central Institute of Mental Health, Mannheim, Germany) were used for the ADE experiments. All animals were housed individually in standard rat cages (Ehret, Emmendingen, Germany) under a 12‐h artificial light–dark cycle (lights on at 7:00 AM). Room temperature was kept constant (temperature: 22°C ± 1°C, humidity: 55% ± 5%). Standard laboratory rat food and water were provided ad libitum throughout the experimental period. Body weights were measured weekly. All experimental procedures were approved by the Committee on Animal Care and Use and carried out in accordance with the local Animal Welfare Act and the European Communities Council Directive of 24 November 1986 (86/609/EEC).

##### 2.2.1.2 Drugs

Alcohol drinking solutions were prepared from 96% ethanol (Merck, Darmstadt, Germany) and then diluted with tap water. Nezavist was suspended by sonicating in vehicle solution of 0.5% methylcellulose/5% Tween‐80 in 0.9% sterile sodium chloride and then diluted with 0.9% sterile sodium chloride to produce the injection volume of 3 mL/kg. The solution was administered intraperitoneally (ip). Control animals received administration of the vehicle solution.

##### 2.2.1.3 Long‐Term Alcohol Self‐Administration With Repeated Deprivation Phases

After 2 weeks of habituation to the animal room, rats were given ad libitum access to water and to 5%, 10% and 20% alcohol (ethanol) solutions (v/v). Spillage and evaporation were minimized by the use of special bottle caps (TSE, Bad Homburg, Germany). With this procedure, the alcohol concentration remains constant for at least 1 week [32]. The positions of bottles were changed weekly to avoid location preferences.

The first 2‐week deprivation period was introduced after 8 weeks of continuous alcohol availability. After the deprivation period, rats were given free access to water and to alcohol solutions for 5 weeks. Then a second 2‐week deprivation period was introduced. This 5‐week alcohol drinking and 2‐week deprivation cycle was performed repeatedly. The long‐term voluntary alcohol drinking procedure including all deprivation phases lasted in total 41 weeks. Prior data [3, 31] demonstrated that this procedure generated animals showing signs of physical dependence upon the initial stages of each deprivation period.

##### 2.2.1.4 Pharmacological Studies

The pharmacological studies were introduced at the end of the fifth or sixth alcohol deprivation period. In order to study the effects of Nezavist, in the first study (20‐mg/kg Nezavist), a group of 14 rats was treated with vehicle, and a group of 12 rats was treated with Nezavist. The mean baseline total alcohol intake, measured over the last 3 days of the free‐choice alcohol consumption period, was approximately the same in both groups (i.e., ~2.5 g/kg/day). In the second study (75‐mg/kg Nezavist), rats were divided into two groups of eight animals each, with mean baseline total alcohol intake of ~2.8 g/kg. After the last day of baseline measurement, the alcohol bottles were removed from the cages leaving the animals with free access to food and water for 20 days in the first experiment and for 14 days in the second experiment. Thereafter, each animal was subjected to a total of 5 intraperitoneal (ip) injections (starting at 7 PM with 12 h intervals) of either vehicle or Nezavist (20 or 75 mg/kg). The alcohol bottles were reintroduced after the second injection (at ~9 AM on the 21st [first experiment] or 15th [second experiment] day of alcohol deprivation) and the occurrence of an ADE was determined. Total alcohol intake (g/kg of body weight/day) and water intake (mL/kg of body weight/day) were measured daily at ~9 AM for three or seven subsequent days for the first and second experiment, respectively. Each rat's body weight was recorded 24 h before the first injection and 12 h after the last injection.

A comparator experiment was performed in which rats were treated with Acamprosate. Rats were divided into two groups of nine animals each, such that the mean baseline alcohol intake was similar for both groups (i.e.,~2.3 g/kg/day). After the last day of baseline measurement, the alcohol bottles were removed from the cages, leaving the animals with free access to food and water for 14 days. Thereafter, each animal received a total of 5 ip injections of Acamprosate (200 mg/kg) or vehicle as described above and in Meinhardt and Sommer [34]. The alcohol bottles were reintroduced after the second injection (at ~9 AM on the first day of re‐exposure). The injections were administered at 12‐h intervals. Alcohol intake (g/kg of body weight/day) and water intake (ml/kg of body weight/day) were measured daily at ~9 AM for a subsequent week.

##### 2.2.1.5 Home Cage Locomotor Activity Measurements by the E‐Motion System

The effect of treatments on locomotor activity was monitored in vehicle‐ and Nezavist‐treated groups. Locomotor activity was monitored from 7 PM to 7 AM starting 3 days before drug treatment, during treatment and for several days posttreatment. Home cage locomotor activity was monitored using an infrared sensor connected to a recording and data storing system (Mouse‐E‐Motion instrument from Infra‐e‐motion, Henstedt‐Ulzburg, Germany). A Mouse‐E‐Motion device was placed above each cage (30 cm from the bottom) so that the rat could be detected at any position inside the cage. The device sampled every second whether the rat was moving or not. The sensor could detect body movement of the rat of at least 1.5 cm from one sample point to the successive one. The data measured by each Mouse‐E‐Motion device were downloaded into a personal computer and processed with Microsoft Excel.

##### 2.2.1.6 Statistical Analysis

Two‐way ANOVA was performed to assess the effect of treatment on total alcohol intake, water intake and alcohol preference between groups and the effect of day within each group, with post hoc pairwise *t*‐tests. Type III testing was performed to account for unbalanced groups with a Mauchly's sphericity correction. All statistical analyses were performed in R (version 4.4.1) using the ‘rstatix’ package. Significance level was set at *p* < 0.05.

To further analyse the ADE results, the amount of each concentration of alcohol (5%, 10% or 20%) consumed by the animals treated with vehicle or 20‐mg/kg (×5) or 75‐mg/kg (×5) Nezavist or Acamprosate was compared. Individual alcohol consumption values that were more than 2 standard deviations from the mean of that treatment group/day/alcohol concentration combination were treated as missing data. A linear mixed model was used to initially examine the three‐way ANOVA (treatment group, day and alcohol concentration) for alcohol consumption when including baseline consumption (mean of last three measures prior to deprivation), Days 1–3 (20‐mg/kg Nezavist) or Days 1–7 (75‐mg/kg Nezavist or Acamprosate) of the alcohol deprivation period. Based on the statistical significance of the three‐ and/or two‐way interactions in this model, the data were stratified by day, and a linear mixed model was used at each day to examine the effects of treatment group, alcohol concentration and their interaction. Means and standard errors for each treatment group/day/alcohol concentration combination were estimated as marginal means from the stratified mixed linear models and post hoc pairwise comparisons between treatment groups at each alcohol concentration were executed. The R package *lme4* (version 1.1‐35.5) [35] was used for the linear mixed models, and the R package *emmeans* (version 1.10.5) [36] was used for estimating marginal means and *post hoc* comparisons (R version 4.4.3).

#### 2.2.2 Alcohol (Ethanol) Vapour Exposure and Operant Self‐Administration by Rats (Laboratory of Drs. Olivier George and Giordano de Guglielmo, University of California San Diego, La Jolla, CA)

##### 2.2.2.1 Animals

Methods for operant alcohol (ethanol) self‐administration and alcohol vapour exposure have been described in detail [37, 38, 39]. Adult male Wistar rats (Charles River, Raleigh, NC), weighing 225–275 g at the beginning of the experiments, were housed in groups of 2–3 per cage in a temperature‐controlled (22°C) vivarium on a 12‐h/12‐h light/dark cycle (lights on at 8:00 PM) with ad libitum access to food and water. All behavioural tests were conducted during the dark phase of the light/dark cycle. All procedures adhered to the National Institutes of Health Guide for the Care and Use of Laboratory Animals and were approved by the Institutional Animal Care and Use Committee of The Scripps Research Institute.

##### 2.2.2.2 Drugs

Alcohol drinking solution 10% (w/v) was prepared by dilution of ethanol 95% (w/v) in water.

Nezavist was dissolved in a vehicle composed of 5% DMSO, 5% Emulphor (Solvay Novecare) and 90% distilled water and injected intraperitoneally (ip) at the doses of 0, 20, 35 and 50 mg/kg/4 mL 30 min before each test session. For oral treatment, Nezavist was dissolved in a vehicle composed of 10% DMSO, 10% Emulphor and 80% distilled water and administered by oral gavage 1 h before the test session, or at longer intervals prior to the test session in order to assess the duration of action of Nezavist.

##### 2.2.2.3 Operant Alcohol Self‐Administration

Self‐administration sessions were conducted in standard operant conditioning chambers (Med Associates, St. Albans, VT). Animals were first trained to self‐administer 10% (w/v) alcohol and water solutions until a stable response was maintained. The rats were subjected to an overnight session in the operant chambers with access to one lever (right lever) that delivered water (FR1). Food was available ad libitum during this training. After 1 day off, the rats were subjected to a 2‐h session (FR1) for 1 day and a 1‐h session (FR1) the next day, with one lever delivering alcohol (right lever). All of the subsequent sessions lasted 30 min, and two levers were available (left lever: water; right lever: alcohol) until stable levels of intake were reached. Upon completion of this procedure, the animals were allowed to self‐administer a 10% (w/v) alcohol solution and water on an FR1 schedule of reinforcement (i.e., each operant response was reinforced with 0.1 mL of the solution).

##### 2.2.2.4 Alcohol Vapour Exposure

Once a stable baseline of alcohol self‐administration was reached, the rats were made dependent by chronic, intermittent exposure to alcohol vapour for 3 weeks. They underwent cycles of 14 h on (blood alcohol levels during vapour exposure ranged between 150 and 250 mg%) and 10 h off, during which time behavioural testing for acute withdrawal occurred (i.e., 6–8 h after vapour was turned off when brain and blood alcohol levels are negligible). In this model, rats exhibit somatic withdrawal signs and negative emotional symptoms reflected by anxiety‐like responses and elevated brain reward thresholds.

##### 2.2.2.5 Operant Self‐Administration During Alcohol Vapour Exposure

Behavioural testing occurred three times per week during the 3‐week alcohol vapour exposure period. The rats were tested for alcohol (and water) self‐administration on an FR1 schedule of reinforcement for 30‐min sessions during acute withdrawal (i.e., 6–8 h after termination of vapour exposure). Once escalation of responding for alcohol occurred, animals were treated with drug or vehicle, and alcohol and water self‐administration were measured. Results are reported as number of responses on either the alcohol or water‐associated lever during this session and the responding during the stable baseline prior to alcohol vapour administration. Another group of animals (controls) was treated similarly but was exposed to normal room air containing no alcohol during the 3‐week period in the inhalation chamber. Operant testing was performed with these animals on the same schedule as the alcohol‐treated rats. Operant self‐administration on an FR1 schedule requires minimal effort by the animal to obtain the reinforcement and was considered a measure of voluntary intake.

##### 2.2.2.6 Statistical Analysis

Data were analysed with one‐ or two‐way ANOVA or *t*‐test. ANOVAs were followed by the Neuman–Keuls test when appropriate. Statistical significance was set at *p* < 0.05.

### 2.3 General Phenotyping and Other Alcohol‐Related Behaviours (Animal Models Core Facility, Scripps Research, Dr. Amanda Roberts, Director) and Lohocla Research Corporation/University of Colorado Anschutz Medical Campus

All animal work was conducted in accordance with the guidelines of the American Association for the Accreditation of Laboratory Animal Care and was approved by the Institutional Animal Care and Use Committee of The Scripps Research Institute (TSRI) or the University of Colorado. In all studies, mice were housed 4/cage and rats were housed 2/cage with ad libitum access to standard laboratory chow and water.

#### 2.3.1 Locomotor Activity (Mice) (TSRI)

Forty male C57BL/6 J mice (Jackson Labs), 10 weeks old at arrival, were used for this test (10 mice per dose group). The light cycle was 8:00 AM off, 8:00 PM on, with testing done during the dark cycle.

Mice were injected ip with vehicle (5% DMSO, 5% Cremophor, 90% physiological saline) or with 50‐, 200‐ or 500‐mg/kg Nezavist. One hour later, locomotor activity was measured for 30 min. Locomotor activity was measured in polycarbonate cages (42 × 22 × 20 cm) placed into frames (25.5 × 47 cm) mounted with two levels of photocell beams at 2 and 7 cm above the bottom of the cage (San Diego Instruments, San Diego, CA). These two sets of beams allowed for the recording of both horizontal (locomotion) and vertical (rearing) behaviour. A thin layer of bedding material was applied to the bottom of the cage. Data were collected in 1‐min intervals.

#### 2.3.2 Elevated plus Maze (Anxiety) (Mice) (TSRI)

Sixty male C57BL/6 J mice (Jackson Labs, ME), 12 weeks old on arrival, were used in this experiment. The mice were housed under reverse light conditions (off 8:00 AM, on 8:00 PM). All testing occurred between 9:00 AM and 1:00 PM.

Mice were randomly assigned to receive vehicle (5% DMSO, 5% Cremophor, 90% physiological saline), 50‐ or 150‐mg/kg Nezavist ip 30 min prior to the initiation of testing in two different elevated plus mazes such that there were 10 mice per group (i.e.,10 mice per dose per maze). Both plus‐maze apparatuses have four arms (5 × 30 cm) at right angles to each other, elevated 30 cm from the floor. In both mazes, two opposite arms have no walls (i.e., they are open). The other two arms are either clear (Clear Enclosed Sides) or are opaque black (Dark Enclosed Arms). Controls tested in the Clear Enclosed Sides apparatus spend 35%–40% of their time on the open arms, allowing changes to be detected bidirectionally, whereas mice tested in the original style plus‐maze (Dark Enclosed Arms) typically spend 10%–15% of their time on the open arms, the small percentages making it difficult to detect anxiogenic‐like effects. Both mazes were used in this experiment to optimize the ability to detect both anxiolytic as well as anxiogenic compound effects. Mice were placed on the center of the maze, and behaviour was videorecorded for 5 min. Decreases in % open arm time, calculated as: 100*open arm time/(open arm time + closed arm time), indicate increased anxiety‐like behaviour, while increases indicate anxiolytic‐like behaviour [40]. Total arm entries are a measure of locomotor activity effects [40].

#### 2.3.3 Incoordination (Rotarod) (Mice) (TSRI)

This study used 40 male C57BL/6 mice (Jackson Labs), 10 weeks old at arrival, with 10 mice per Nezavist dose group. The study was performed during the dark cycle (8:00 AM to 8:00 PM). Rotarod balancing requires a variety of proprioceptive, vestibular and fine‐tuned motor abilities as well as motor learning capabilities [41]. A Roto‐rod Series 8 apparatus (IITC Life Sciences, Woodland Hills, CA) was used. For training and testing, an accelerating test strategy was used whereby the rod started at 0 rpm and then accelerated by 10 rpm for each additional minute. When an animal dropped onto the individual sensing platforms below the rotating rod, the time from placement on the rotarod (‘latency to fall’) was used to calculate the speed (rpm) at which the mouse could no longer stay on the rotarod. The mice were trained 6 times per day in two sets of three trials, with 1 min between each trial within a set and approximately an hour between each set. For testing, mice were injected ip with vehicle (5% DMSO, 5% Cremophor, 90% physiological saline), 50‐, 200‐ or 500‐mg/kg Nezavist. The speed at which the animals fell was recorded at 0 min (baseline, predose), 30 min after injection and 120 min after injection (three sessions at each time point).

#### 2.3.4 Forced Swim Test (Rats) (Lohocla Research Corporation; University of Colorado)

Sixteen (eight male and eight female) adult Sprague–Dawley rats were used for these experiments. The characteristic behaviour of the test, termed immobility, develops when a rodent is placed in a tank of water for a period of time in which it cannot escape, stops attempting to escape and begins to make only the movements required to balance the body and float with its head above the water [42].

The development of immobility is facilitated by a 15‐min pretest administered 24 h before the 5‐min actual test. The latency to become immobile and the duration of immobility decrease when antidepressants are administered between the pretest and the test. Nezavist (50 mg/kg) or vehicle (5% DMSO, 5% Cremophor and 90% physiological saline) was administered by ip injection 90 min prior to the 5‐min test.

Forced swim sessions were conducted by placing the animal individually in a large plastic cylindrical chamber (45 × 20 cm) containing 23°C–25°C water that is approximately 30 cm deep. The water is at a height such that the animal cannot escape or touch the bottom of the chamber. On Day 1, the animal is placed in the cylinder for 15 min. On Day 2, ~24 h later, a 5‐min test is administered. The latency to start floating and the amount of time spent trying to escape are measured. The 5‐min test on Day 2 is video recorded and scored using Noldus Ethovision and/or by an observer that is blind to the animal group or treatment. At the end of the swim session, the animals are towel dried and placed in a clean cage warmed with either a heat lamp or heating pad. The water in the test arena is changed between each subject.

#### 2.3.5 Interaction With Ethanol (Alcohol) (Loss of Righting Reflex) (Mice) (TSRI)

Eighteen male C57BL/6 J mice (Jackson Labs), 10 weeks old at arrival, were used for this study, with six mice in each Nezavist dose group. The light cycle was 8:00 AM on, 8:00 PM off, with testing during the dark cycle. Mice were injected ip with vehicle (5% DMSO, 5% Cremophor, 90% physiological saline), 50‐ or 200‐mg/kg Nezavist. Thirty minutes later, mice were injected ip with 3.5 g/kg 95% ethanol (20% v/v). At 1 min after ethanol treatment, and every 3 min until recovery, mice were assessed for the righting reflex: the mouse was turned on its back in a v‐shaped apparatus and watched for turning over. If this occurred within 5 s and also occurred in a second immediate test, the mouse was determined to have regained its righting reflex. At this point, retro‐orbital blood sampling took place for determination of blood alcohol level.

#### 2.3.6 Interaction With Ethanol (Alcohol) (Incoordination) (Mice) (TSRI)

Eighteen male C57BL/6 J mice (Jackson Labs), 10 weeks old at arrival, were used for this study, with six mice in each Nezavist dose group. The light cycle was 8:00 AM off, 8:00 PM on, with testing taking place during the dark cycle. For these studies, an accelerating Rotarod test strategy was used, starting at 0 rpm and then accelerating by 10 rpm each minute. The mice were trained six times per day in two sets of three sessions, with 1 min between each trial within a set and 1 h between each set. All mice were capable of staying on the Rotarod for 30 s at 7 rpm, which was chosen for the test. On the test day, mice were tested to confirm that they could remain on the Rotarod for 30 s at 7 rpm and were then injected ip with vehicle (5% DMSO, 5% Cremophor, 90% physiological saline), or 50‐ or 200‐mg/kg Nezavist. Twenty minutes after Nezavist treatment, mice were tested on the rotarod to confirm that they could stay on the Rotarod for 30 s at 7 rpm. Ten minutes later, mice were injected ip with 1.5 g/kg of 95% ethanol (20% v/v) and were tested again after 1 min and every 3 min until they could again maintain balance for 30 s at 7 rpm. At this point, retro‐orbital blood was obtained for blood alcohol level determination.

#### 2.3.7 Measurement of Blood Alcohol (Ethanol) Levels (Mice) (TSRI)

Blood (retro‐orbital) was collected in capillary tubes and emptied into Eppendorf tubes containing evaporated heparin and kept on ice. Samples were centrifuged, and plasma decanted into fresh Eppendorf tubes. The plasma was then injected into an oxygen‐rate alcohol analyser (Analox Instruments, Lunenburg, MA) for blood alcohol determination. Five pairs of ethanol standards (50–300 mg%) were run before the samples.

#### 2.3.8 Conditioned Place Preference (Mice) (TSRI)

Forty male C75BL/6 J mice (Jackson Labs; ~10 weeks old at arrival) were used in the (Experiment 1) study of Nezavist and 30 CF‐1 mice were used in the (Experiment 2) study of the Nezavist metabolite, DCUKA, in comparison to morphine (positive control). Animals were housed with a light cycle of 8:00 AM off and 8:00 PM on, and testing occurred during the dark cycle.

The place conditioning apparatuses consisted of two connected compartments of equal size (dimensions of entire apparatuses: 44 × 22 × 22 cm) separated by doorways (4 × 4 cm) or closed off from each other completely. Two floor textures that have been generally shown to be equally preferred by C57BL/6 J mice were used such that each chamber had one compartment with each floor type. For the 30‐min baseline pretest (Day 1), mice were allowed to explore both compartments freely. For place conditioning (Days 2–7), mice showing no bias for one compartment in the pretest received drug or vehicle and immediately were confined to one randomly determined chamber for 30 min. On alternate days the treatment was reversed, as was the compartment in which the mouse was placed. This 2‐day sequence was repeated three times for a total of 6 days of injections, three each of drug and saline. Treatment order and the compartment used for drug pairing were counterbalanced. For the test, mice were again allowed to explore both compartments freely with no injections given. The time spent in each compartment (all four paws in) was determined on Days 1 and 8. These tests were videotaped and scored by a technician blinded to treatment conditions.

In Experiment 1, at 0 min on each of these days, animals were injected (ip) with vehicle (5% DMSO, 5% Cremophor, 90% physiological saline), 50‐, 100‐ or 200‐mg/kg Nezavist (*n* = 10 animals/group). In Experiment 2, at 0 min on each of these days, animals were injected (ip) with morphine (10 mg/kg) or DCUKA (50 or 150 mg/kg). Fifteen minutes after dosing, animals were placed in the appropriate side of the place conditioning box for 30 min. On Day 8, animals underwent 30‐min place conditioning testing with no injection.

#### 2.3.9 Statistical Analysis

Data were analysed by ANOVA and Fishers PLSD post hoc tests.

### 2.4 Pharmacokinetic Studies of Nezavist and Its Initial and Primary Metabolite DCUKA in Rats

#### 2.4.1 Measurement of Blood and Brain Nezavist and DCUKA Levels (Lohocla Research Corporation Contract with Preclinical Research Services, Fort Collins CO and University of Colorado Cancer Center Pharmacology Shared Resource, Colorado State University, Fort Collins, CO)

All studies were performed in accordance with the guidelines of the American Association for the Accreditation of Laboratory Animal Care and were approved by the Institutional Animal Care and Use Committee of Preclinical Research Services. Several experiments were performed to evaluate concentrations of Nezavist in the blood and brain. In the first experiment, adult male Sprague–Dawley rats (six per group) were injected ip with Nezavist in a vehicle of 5% DMSO, 5% Kolliphor EL and 90% physiological saline. One group received a single dose of 50 mg/kg of Nezavist; a second group received three doses of 50 mg/kg at 2‐h intervals; a third group received a single dose of 400 mg/kg of Nezavist. Blood samples were obtained predose and at 15, 30, 60, 90, 120, 150, 180, 210, 240 and 300 min after dosing (after the last dose when multiple doses were given). Animals were euthanized and brains were collected at the last time point for blood collection as described below. In a second experiment, groups of male Sprague–Dawley rats (nine per group) were treated with a 50‐mg/kg single dose of Nezavist or 3 × 50 mg/kg dose of Nezavist ip as above. Brains were collected as described below (three animals per group per time point) at 1, 2 or 3 h after dosing. Blood and brains were collected 1, 2.5 and 5 h (four animals per time point) after Nezavist administration, as described below. In all experiments, whole blood was collected from animals via the jugular vein and stored in microtainer blood collection tubes containing lithium heparin as the anticoagulant at −70°C. For brain collection, animals were euthanized by CO_2_ inhalation and whole brains were removed, snap frozen and stored at −70°C. Prior to quantification, brain samples were homogenized with water to achieve a final protein concentration of 100 mg/mL.

Nezavist and DCUKA (the initial and primary metabolite of Nezavist) concentrations in whole blood and brain were quantified by liquid chromatography‐tandem mass spectrometry (LC–MS/MS) using DCUK‐OMe as internal standard. For whole blood samples, the lower limit of quantitation was 1 ng/mL for Nezavist and 5 ng/mL for DCUKA. For rat brain, the lower limit of quantitation was 5 and 10 ng/g for Nezavist and DCUKA, respectively.

#### 2.4.2 Additional Measures of Blood and Tissue Nezavist and DCUKA Levels in Rats (Lohocla Research Corporation Contract With Charles River Laboratories (CRL))

All studies were performed in accordance with the guidelines of the American Association for the Accreditation of Laboratory Animal Care and were approved by the CRL Institutional Animal Care and Use Committee. Male Cesarian Derived (Sprague–Dawley) rats fitted with indwelling jugular vein catheters (JVC), 226–250 g upon arrival were orally administered 50‐ or 150‐mg/kg Nezavist prepared in a vehicle of 5% DMSO, 5% Emulphor and 90% sterile water.

Following Nezavist administration, whole blood was collected at 30‐, 60‐, 90‐ and 120‐min postdose and stored in NaF (anticoagulant) collection tubes. After whole blood collection, animals were humanely euthanized and liver, and brain tissues were collected at 30, 60 and 120‐min (*n* = 3 animals per time point), mixed with NaF and homogenized. Nezavist and DCUKA concentrations in whole blood and tissue (liver and brain) were quantified by LC–MS/MS using deuterated internal standard. The lower limit of quantitation (LLOQ) for Nezavist in whole blood, liver and brain was 1.00 ng/mL, 3.00 ng/g and 7.50 ng/g, respectively. DCUKA concentrations were determined through back calculation against the Nezavist curve.

Statistical analyses including regression analysis and descriptive statistics including arithmetic means and standard deviations, accuracy and precision were performed using Analyst v1.6.2 from MDS Sciex and Microsoft Excel.

#### 2.4.3 Measures of Plasma Nezavist and DCUKA Levels After Administration of Nezavist Spray‐Dried Dispersion (SDD) in Rats (Lohocla Research Corporation Contracts With PreClinical Research Services Inc. (PCRS) and Sekisui XenoTech LLC)

Animal usage was reviewed and approved by the PCRS Institutional Animal Care and Use Committee for compliance with regulations prior to study start. Animal welfare for this study was in compliance with The USDA Animal Welfare Act and The Guide for the Care and Use of Laboratory Animals and the American Veterinary Medical Association (AVMA) Guidelines for Euthanasia. Male Wistar rats, age‐matched with a body weight of 268.23–305.85 g at the time of dosing, were used for this study. Rats were orally administered 250 mg/kg of Nezavist in a 20% loading Nezavist: HPMCAS‐MG spray‐dried dispersion in HPMC suspension.

PK blood samples were collected t predose and at 0.25‐, 0.5‐,1‐, 1.5‐, 2‐, 4‐, 8‐ and 12‐h postdose. Blood (200 μL) was collected into NaF lined microtubes (RAM Scientific, 200‐μL Sodium Fluoride Capillary Collection Tubes, Item 07 7340) via the capillary tube attached to the cap. The filled microtubes were inverted several times to allow the mixing of the NaF with the whole blood. The blood samples were centrifuged at 2°C–8°C for 10 min at approximately 3000 rpm. Plasma was then harvested and stored in labelled cryovials. The samples were stored at −70°C until shipment to Sekisui XenoTech LLC, where Nezavist and DCUKA concentrations in plasma were quantified by LC–MS/MS using deuterated internal standard.

### 2.5 Effect of Nezavist on Intestinal Motility (Laboratory of Dr. Jerome Swinny, University of Portsmouth, UK)

All animal procedures were approved by the Animal Welfare and Ethics Board of the University of Portsmouth and were performed in accordance with the Animal (Scientific Procedures) Act,1966 (UK).

Nezavist was dissolved in dimethyl sulfoxide (DMSO). The final concentration of DMSO in the bath had no effect on the amplitude or frequency of spontaneous muscle contraction. Concentrations of Nezavist ranged from 300 nM to 100 μM.

The effect of Nezavist on the mouse ileum and colon was examined by previously described methods [43, 44]. Male C57BL/6 mice were obtained from the University of Portsmouth Bioresource Center and had ad libitum access to standard chow and water. Mice were euthanized by cervical dislocation and segments of the intestine (ileum and distal colon) were collected from mice, placed in physiological solution containing (in mM) NaCl 140, NaHCO_3_ 11.9, D + glucose 5.6, KCl 2.7, MgCl_2_.6H_2_O 1.05, NaH_2_PO_4_.2H_2_O 0.5, CaCl_2_ 1.8 and warmed to 32°C. Intraluminal contents were removed by gentle flushing with physiological solution. Approximately 2‐cm‐long segments were mounted in a Harvard organ bath (10‐mL chamber) filled with the physiological solution bubbled with 95% O_2_/5% CO_2_ gas. Contractile activity for each intestinal tissue strip was recorded using an isometric force transducer. The tissue was placed under 1 g of resting tension and allowed to equilibrate for 30 min. Baseline measurements were used to quantify the force of basal tone. After a stable baseline was established, Nezavist was added to the bath, and the tissue was allowed to reach maximum response. Ten‐minute epochs before and after the drug additions were used for quantification of the drug‐induced changes in the force and frequency of spontaneous contractions. One piece of tissue was used per animal (*n* = 5 animals/condition). The frequency and amplitude (force) of individual spontaneous contractions were determined before and after the drug exposure.

#### 2.5.1 Statistical Analysis

Data were analysed by ANOVA and Tukey post hoc testing.

### 2.6 Effect of Nezavist on Vagal Firing (Laboratory of Dr. Wolfgang Kunze, McMaster University, Ontario, Canada)

The experiments on the effects of Nezavist on vagal nerve activity were performed using methods described in detail in West et al. [45]. All experiments were carried out in accordance with the guidelines of the Canadian Council on Animal Care and ARRIVE Guidelines and were approved by the McMaster University Animal Research Ethics Board.

Nezavist was dissolved in DMSO to make a stock solution. The stock solution was diluted in Krebs buffer (118‐mM NaCl, 4.8‐mM KCl, 25‐mM NaHCO_3_, 1.0 NaH_2_PO_4_, 1.2‐mM MgSO_4_, 11.1‐mM glucose and 2.5‐mM CaCl_2_ bubbled with 95% O_2_–5% CO_2_ (‘carbogen’)) to concentrations of either 10‐ or 100‐μM Nezavist. The final concentration of DMSO was ≤ 1%, which had no effect on vagal nerve firing.

Adult male C57BL/6 mice were obtained from Charles River (Montreal) and had ad libitum access to standard chow and water. Mice were euthanized by cervical dislocation. Segments of the jejunum were collected with an attached mesenteric arcade containing a neuromuscular bundle and placed in Krebs buffer. An ex vivo mouse intestinal segment perfusion preparation was used to record afferent single unit vagal activity [46, 47, 48] (Figure 10A) before and after exposure of the gut lumen to Nezavist or Krebs buffer. The gut segment was placed onto the stage of an inverted microscope and the lumen gravity perfused at 1 mL/min with room temperature (22°C) carbogenated Krebs or Krebs plus one of the luminal additives using several Mariotte bottles. The serosal compartment was separately perfused at 5 mL/min with Krebs solution to which 3‐μM nicardipine had been added to isolate vagal chemosensory responses by preventing active muscle contractions but not vagal responses to gut distension.

To record afferent vagal nerve activity, the cleaned nerve from the tissue segment was sucked into a glass recording pipette that was attached to a patch‐clamp electrode holder and extracellular nerve recordings were made by running pClamp software using a Multi‐Clamp 700B amplifier and Digidata 1440A signal converter (Molecular Devices, LLC. 3860 N First Street San Jose, CA 95134). Baseline recordings in the gut lumen were performed for 15 min with Krebs buffer. Following this, the luminal perfusate was switched for 40 min to one containing Krebs buffer with Nezavist added (either 1, 10 or 100 μM). The effects of GABA_A_ receptor antagonists (50‐μM picrotoxin or bicuculline) or the nicotinic cholinergic antagonist, mecamylamine (50 μM) on the response to 100‐μM Nezavist were also determined. Then the perfusate was again switched to Krebs buffer and recording continued for 30 min. Single units (belonging to an individual vagal fibre) were discriminated by their action potential shape, amplitude and width in response to cholecystokinin, using a dedicated programme for extracellular single unit action potential analysis (Dataview written by Dr. W. J. Heitler, School of Psychology and Neuroscience, University of St Andrews Scotland, UK). Single unit events were subdivided into vehicle (Krebs) and treatment periods, and for each event, mean interspike intervals (MII) were recorded. In some experiments, comparing the effects of diazepam, cholecystokinin (CCK) or ethanol to Nezavist, other parameters (gap duration [GD], burst duration [BD] and intraburst interval [IBI]) were also recorded [45].

#### 2.6.1 Statistical Analysis

MII in the presence of Krebs (vehicle) or drugs were compared by paired *t*‐test. MII paired differences (drug MII response—Krebs MII response) were compared by effect size as given by the partial eta squared statistic (*η*2p) between concentrations of Nezavist. For interpreting *η*2p, 0.01 indicates a small, 0.06 a medium and 0.14 a large effect size. Fractional differences ((Treatment‐Krebs)/Krebs) were compared by unpaired *t*‐test.

### 2.7 Effect of Nezavist on c‐Fos Levels in Rat Nucleus Tractus Solitarius (NTS) (Laboratory of Drs. Howard Becker and Christina LeBonville, Medical University of South Carolina, Charleston, South Carolina)

#### 2.7.1 Animals and Final Group Sizes

All animal procedures were approved and facilities inspected by the Medical University of South Carolina (MUSC) Institutional Animal Care and Use Committee (IACUC) in accordance with the guidelines established by the US National Research Council.

Adult male C57BL/6 J mice (*N* = 80, 9 weeks old) were purchased from Jackson Laboratories (JAX Stock #000664, Bar Habor, ME). Mice were singly housed with ad libitum access to food and water under a 12‐h light/dark cycle (light on at 02:00, off at 14:00). All experimentation occurred during the light part of the light/dark cycle. Mice were first treated with an intraperitoneal (ip) injection of lipopolysaccharide (LPS; 1 mg/kg) or vehicle followed 30 min later by an ip injection of Nezavist (100 mg/kg) or placebo. Mice were sacrificed at two time points, 90‐ and 150‐min post‐Nezavist/placebo administration. Thus, this study produced eight groups, based on a 2 (Drug or Placebo) × 2 (LPS or Vehicle) × 2 (90 or 150 min) factorial design, with 10 mice per group. Mice were run in four cohorts spread across 4 days (within 2 weeks), with groups divided equally across days. Of the 80 brains collected, 21 were lost due to technical issues with tissue processing or imaging. The final data, therefore, were from *N* = 59 mice (4–10 per group).

#### 2.7.2 Drugs

Lipopolysaccharide (LPS) was obtained from Sigma‐Aldrich (L3024, *Escherichia coli* serotype O111:B4), dissolved in 0.9% sterile saline to a 5‐mg/mL concentration and stored as 1‐mL aliquots at −80°C until use. One day prior to experimentation, frozen LPS aliquots were thawed, diluted 1:10 with saline to a working 0.1‐mg/mL solution and stored at 4°C overnight. The working LPS solution was allowed to come to room temperature for ip injection (10 mL/kg) of a 1‐mg/kg dose. Sterile saline ip injections (10 mL/kg) were given to vehicle control groups. Nezavist powder was supplied by Lohocla Research Corporation (Aurora, CO, USA). Immediately before experimentation, 150 mg of Nezavist was dissolved in 1.5 mL 100% DMSO (Sigma‐Aldrich). Then 1.5 mL of Cremophor EL (Calbiochem, USA) was added and the solution was diluted to 30 mL with double‐distilled water for a final 5‐mg/mL Nezavist suspension in 5% DMSO and 5% Cremophor. The working Nezavist suspension was vortexed just before drawing up each syringe for ip injection in a 20‐mL/kg volume. The final dose of Nezavist administered was therefore 100 mg/kg. Placebo groups received ip injections (20 mL/kg) of 5% DMSO and 5% Cremophor in double‐distilled water.

#### 2.7.3 c‐Fos Immunohistochemistry

At either 90 or 150 min after Nezavist/placebo treatment, mice were deeply anaesthetized with urethane (1.5 mg/kg, ip) and then transcardially perfused for 2‐min with 1X phosphate‐buffered saline (PBS; pH 7.4) and then for 3 min with 4% formaldehyde (paraformaldehyde dissolved in 1X PBS) at a 25‐mL/min flow rate. Brains were removed, postfixed in 4% formaldehyde overnight at 4°C and then placed into 30% (w/v) sucrose until fully saturated (sunk to the bottom of tube) prior to flash freezing in a 2‐methylbutane dry ice bath. Frozen brains were stored at −80°C in aluminium foil until sectioned. Coronal sections (40 μm) were cut on a cryostat (Microm/Thermo Fisher Scientific, HM525, Waltham, MA USA) and collected into cryopreserve solution (1% (w/v) polyvinylpyrrolidone (PVP, MilliporeSigma, Burlington, MA, USA) and 50% (v/v) ethylene glycol (ThermoFisher Scientific) in 1X PBS) for −20°C storage prior to staining. c‐Fos immunohistochemistry was carried out on free‐floating sections in staining nets (Brain Research Laboratories) balanced across all independent variables. All incubations and rinses took place at room temperature on an orbital shaker. After rinsing stored tissue, sections were first placed into 0.3% H_2_O_2_ for 15 min to quench endogenous peroxidases, followed by blocking in 5% normal goat serum in 1X PBS with 0.3% TritonX‐100 (PBST) for 1 h. Sections were washed in 1X PBST between all steps, with the exception of the final washes which were in PBS. Primary antibody (1:4000 guinea pig anti‐c‐Fos, Synaptic Systems, 266 308) diluted in blocking solution was incubated with tissue overnight. Sections were then incubated in goat antiguinea pig biotin‐conjugated secondary antibody (1:1000, Jackson Immuno Research, 106‐065‐003) for 1 h and ABC‐HRP (Vector Elite Kit, Vector Laboratories Inc., Newark, CA, USA) as directed for 45 min. The reaction was visualized via incubation for 5 min in 0.05% 3,3′‐diaminobenzidine (DAB), 0.05% nickel ammonium sulphate and 0.0015% H_2_O_2_. The tissue was then mounted onto Superfrost Plus slides (Thermo Fisher Scientific), dried and counterstained with Gill's haematoxylin No. 1 (MilliporeSigma, GHS132). Slides were coverslipped using Permount mounting medium (Thermo Fisher Scientific).

#### 2.7.4 Image Analysis

Brightfield images were captured at 20X magnification using a ZEISS epifluorescence microscope (Axioscope 5, ZEISS AG, Oberkochen, Germany). c‐Fos‐positive neurons were counted bilaterally from sections taken across the anterior–posterior axis of the NTS from AP coordinates −6.48 to −7.72 mm relative to bregma [49]. Fos‐positive nuclei were manually labelled across the entire image using the multi‐point tool and counted using the measure feature in ImageJ software [50]. Labelling, counting and image quality control were conducted blind to treatment groups. Images were classified as either anterior or posterior based on the appearance of the area postrema (approximately AP −7.32 mm). NTS rostral to the level of the area postrema was classified as anterior NTS, while posterior NTS coincided with the area postrema. One image was removed from the final dataset due to being a statistically significant upper outlier (Grubbs Test, *p* < 0.0001) whose count was more than 100 cells away from the nearest data point. The final dataset consisted of 526 images, 1–18 images per subject.

#### 2.7.5 Statistical Analysis

Since there were different amounts of images from each subject, the c‐Fos‐positive cell counts were analysed using a linear mixed model with LPS/Vehicle (Drug 1), Nezavist/Placebo (Drug 2) and rostral/caudal (Position) as fixed factors and mouse as a random intercept. Linear mixed models are designed to account for repeated measures even with missing data or unbalanced designs [51] and, thus, were the optimal statistical approach with these data. Helmert contrasts were used for modelling, so while all factors only had two levels, this ensured the contrasts were centred. All tests were performed using Restricted Maximum Likelihood (REML) estimation in R [52] with custom scripts (available upon request), the ‘lme4’ package [35] and guidance from West et al. [51]. Significant interactions were probed with multiple‐comparison adjusted post hoc tests using ‘emmeans’ and ‘stats’ R packages [36, 52]. Final models were evaluated for multicollinearity using generalized variance‐inflation factors (GVIFs) from the ‘car’ R package [53] where GVIFs < 5 were considered to have no issues (all GVIFs were < 2). Degrees of freedom for *t*‐statistics were estimated with the R package ‘lmerTest’ [54] using Satterthwaite approximations, which produce acceptable Type I error rates [55]. Data were visualized using R package ‘ggplot2’ [56] with numerous add‐on packages.

### 2.8 Effect of Nezavist on Peripheral and Hippocampal Cytokine Levels, corticotropin releasing factor (CRF) Levels and Microgliosis (Lohocla Contract with ScanTox Neuro GmbH, Vienna, Austria)

#### 2.8.1 Animals and Regulations

Forty male C57BL/6JRj mice (8–9 weeks old) were obtained from Janvier Labs (Le Genest‐Saint‐Isle, France) and transferred to the Scantox Neuro animal facility. After a general health check and registration, the animals were habituated at standard housing conditions for at least 1 week before treatment. Animals were housed in ventilated cages on standardized rodent bedding. Each cage contained a maximum of five mice. The temperature in the animal room was maintained between 20°C and 24°C, and the relative humidity was maintained between 45% and 65%. Animals were housed under a constant light‐cycle (12 h light/dark). Dried, pelleted standard rodent chow (Altromin) and normal tap water were available to the animals ad libitum.

The study was performed according to Scantox Neuro's Global Quality Policies as implemented in Scantox Neuro's current internal SOPs considering GxP requirements. The Scantox Neuro animal facility was fully accredited by the Association for Assessment and Accreditation of Laboratory Animal Care (AAALAC). All procedures in this study were approved by the Animal Care and Welfare Committee.

#### 2.8.2 Additional Regulations and Laws That Applied for Animal Welfare

- Austrian Animal Experiments Regulation: Verordnung des Bundesministers für Wissenschaft und Forschung zur Durchführung des Tierversuchsgesetzes 2012 (Tierversuchs‐Verordnung—TVV 2012), BGBl. II Nr. 542/2020
- Austrian Animal Experiments Law: Bundesgesetz über Versuche an lebenden Tieren (Tierversuchsgesetz 2012—TVG 2012) BGBl. I Nr. 76/2020
- Austrian Animal Welfare Law: Bundesgesetz über den Schutz der Tiere (Tierschutzgesetz—TSchG) BGBl. I Nr. 130/2022
- Directive 2010/63/EU of the European Parliament and of the Council of 22. September 2010 on the protection of animals used for scientific purposes; Consolidated version 26.06.2019

Furthermore, the study was performed according to the regulations of the Austrian Genetic Engineering Law (BGBl. I Nr. 8/2022). Safety precautions operating within the test facility were applied to the study.

#### 2.8.3 Nezavist Preparation

The solid compound was formulated in vehicle (5% DMSO/5% Cremophor EL in distilled water) to produce a suspension with a final dosing concentration of 5 (low dose) or 10 mg/mL (high dose) for intraperitoneal injection at 10 mL/kg.

1. Solid compound was dissolved in pure DMSO.
2. Cremophor EL was added and mixed thoroughly.
3. Sufficient distilled water was added slowly with mixing to produce a final suspension containing 5‐mg/mL (low dose) or 10‐mg/mL (high dose) Nezavist, with a final DMSO concentration of 5% and a final Cremophor EL concentration of 5%.

Dosing formulations for the high and low dose were freshly prepared separately (NOT by dilution of the higher dose) and used for a maximum duration of 36 h. During treatments, the dosing formulations were kept at room temperature and were constantly stirred to ensure that a homogenous suspension was drawn up into the syringe. For storage, dosing formulations were kept refrigerated at 2°C–8°C and protected from light and were warmed to room temperature with vigorous mixing.

#### 2.8.4 LPS Preparation

Lyophilized LPS was reconstituted in endotoxin‐free water to obtain a 5‐mg/mL stock solution (vortexed until completely solubilized). The 5‐mg/mL stock solution was aliquoted and stored at 4°C for short term storage or at −20°C for long term storage. On treatment days, the 5‐mg/mL stock solution was diluted 1:50 in endotoxin‐free water to a final dosing concentration of 0.1 mg/mL for ip injection at 5 mL/kg.

#### 2.8.5 Experimental Overview and Treatment

After habituation, animals were randomly allocated into four groups (A‐D) with *n* = 10 animals/group as shown below:

#### 2.8.6 Group allocation

**Table:** 

Group	*n*=	Genotype	Sex	Age at start (weeks)	Vehicle or LPS treatment	Test item
A	10	C57BL/6JRj	M	11	Vehicle (H_2_O) daily ip for 4 d	Nezavist vehicle daily ip for 4 days
B	10	C57BL/6JRj	M	11	LPS (0.5 mg/kg) dai LPS (0.5 mg/kg) daily ip for 4 d	Nezavist vehicle daily ip for 4 days
C	10	C57BL/6JRj	M	11	LPS (0.5 mg/kg) daily ip for 4 d	Nezavist (50 mg/kg) daily ip for 4 days
D	10	C57BL/6JRj	M	11	LPS (0.5 mg/kg) daily ip for 4 d	Nezavist (100 mg/kg) daily ip for 4 days

The mice received a daily intraperitoneal (ip) injection with vehicle (endotoxin‐free H_2_O) or LPS (0.5 mg/kg; application volume:5 mL/kg), followed by an additional ip treatment with either Nezavist vehicle (5% DMSO/5% Cremophor EL in distilled water) or Nezavist at two different concentrations (50 or 100 mg/kg; application volume: 10 mL/kg) for four consecutive days.

The injection with Nezavist vehicle or Nezavist (50 or 100 mg/kg) was performed 30 ± 5 min after LPS administration on each day. Body weights were determined once prior to the first treatment, and all treatments were applied based on the animals' actual body weight on the first treatment day.

After each LPS treatment, the mice were placed under an infrared‐light heat lamp to counteract the hypothermic effects of LPS. For all treatment groups, clinical signs (including daily recording of body weight) and termination criteria were monitored daily for a total of 4 days, starting on treatment Day 1 until Day 4, and special care measures (e.g., provision of wet food) were applied if necessary.

On Day 4, all mice were tested for general locomotion in the Open‐Field test (5‐min testing). Behavioural testing was performed in the afternoon, 1 h ± 5 min after receiving the last injection with Nezavist vehicle or Nezavist. On Day 5, all animals were sacrificed in the morning, 18 h ± 10 min after the last LPS treatment, and terminal blood and brain samples were collected.

#### 2.8.7 Behavioural Analysis: Open‐Field Test

The Open‐Field test was performed on Day 4 in the afternoon, 1 h ± 5 min after receiving the last injection with Nezavist vehicle or Nezavist. Spontaneous activity was assessed in the Open Field by evaluating the following parameters: activity [s], distance [m], rearings [s], rearings [n] and thigmotaxis [s]. For that purpose, an opaque Open‐Field Box (45 × 45 × 23.5 cm) in combination with a computerized video tracking system (Noldus EthoVision XT 14) was used.

The mice were brought to the room at least 45 min before the start of the testing. Each test session lasted for 5 min to check the mice's behaviour in the new surroundings, as the first minutes of the Open‐Field test were the most suitable to display the exploratory behaviour of the animals. After the testing session, the number of faecal boli was counted, as a measure of emotionality. The Open Field was cleaned with 70% isopropanol after each mouse to eliminate odour traces. Testing was performed under standard room lighting conditions during the light phase of the circadian cycle.

#### 2.8.8 Terminal Blood Sampling and Plasma Preparation

Mice were euthanized by injection of Pentobarbital (600 mg/kg, dosing 10‐μL/g body weight). The thorax was opened and blood was collected by heart puncture with a 23‐gauge needle. The needle was removed and the blood was transferred to the MiniCollect K_2_EDTA (potassium ethylenediaminetetraacetic acid) sample tube. The tube contents were mixed thoroughly to facilitate homogeneous distribution of the EDTA to prevent clotting. The blood samples were centrifuged at 3000 ×*g* for 10 min at room temperature (22°C). Plasma was transferred to pre‐labelled 1.5‐mL *LoBind* Eppendorf tubes (total of 2 aliquots per animal, 1 × 60 μL + rest), frozen on dry ice and stored at −80°C.

#### 2.8.9 Perfusion

Animals were transcardially perfused with 0.9% saline. A 23‐gauge needle connected to a bottle with 0.9% saline was inserted into the left ventricle. The thoracic aorta—between the lungs and the liver—was clamped with hemostatic forceps to block the flow from the heart to the abdomen but allowing the flow to the brain. The right atrium was opened with scissors. A constant pressure of 100 to 120 mmHg was maintained on the perfusion solution by connecting the solution bottle to a manometer‐controlled air compressor. Perfusion was continued until the skull surface turned pale, and only perfusion solution instead of blood was exiting from the right atrium.

#### 2.8.10 Brain Sampling

After perfusion, the skull was opened and the brain was removed carefully and hemisected on a cooled surface. The left hemibrain was further dissected on a cooled surface into *hippocampus* and the *rest of the brain*. All collected parts were weighed, snap frozen on dry ice and stored at −80°C. In total, *n* = 40 hippocampal samples and *n* = 40 rest brain samples were collected. The right hemibrain was fixed by immersion in freshly prepared 4% paraformaldehyde in phosphate buffer (PB; pH = 7.4) for 2 h at RT.

#### 2.8.11 Histology

##### 2.8.11.1 Tissue Preparation

Following fixation by immersion in freshly prepared 4% paraformaldehyde in phosphate buffer (PB; pH 7.4) for 2 h at RT, right hemibrains of all animals (total *n* = 40) were then transferred to 15% sucrose/PBS and stored at 4°C until the sample sank to the bottom of the tube to ensure cryoprotection (usually overnight). Tissue blocks were then trimmed as needed, transferred to cryomolds, embedded in OCT medium, frozen in dry ice‐cooled isopentane and stored at −80°C.

##### 2.8.11.2 Sectioning

All frozen brain samples (total *n* = 40) were sectioned sagittally at 10 μm thickness on a Leica CM1950 or a Thermo Scientific NX70 cryotome, using the following section scheme:

Five consecutive cryosections were collected and the next 25 sections per level were discarded. This collection scheme was repeated for 12 levels. In total 12 × 5 = 60 sections were collected (total of 2400 sections from 40 mice). Sectioning levels were chosen according to the brain atlas [49]. Collection of sections started at a level ~0.2‐mm lateral from midline and extended through the hemisphere, in order to ensure systematic random sampling through the target region (hippocampus). Sections were stored at −20°C.

##### 2.8.11.3 Immunofluorescence

For each incubation a uniform systematic random set of five sections per mouse was selected (one section each from Levels 2, 4, 6, 8 and 10); for information on systematic random sampling follow this link: http://www.stereology.info/sampling/.

Histological labelling experiments were executed on these sets of sections: Microgliosis (Iba1) and astrocytosis (GFAP) as well as CD68‐positive cells were evaluated on sections of all processed brains (*n* = 40 brains; 200 sections total) using triple immunofluorescent labelling with primary antibodies:

Guinea pig anti‐Iba1 monoclonal antibody ([Gp311H9], Synaptic Systems GmbH, 234 308; Scantox #847)

Rabbit anti‐GFAP polyclonal antibody (DAKO, Z0334; Scantox #29)

Rat anti‐CD68 monoclonal antibody [FA‐11] (BioRad, MCA1957; Scantox #136)

All sections were counterstained with the nuclear dye DAPI. Binding of primary antibodies was visualized using the following highly cross‐absorbed secondary antibodies:

Donkey antiguinea pig IgG H + L Cy3‐conjugated (Jackson Immunoresearch)

Donkey antirabbit IgG H + L AlexaFluor 750‐conjugated (Abcam)

Donkey antirat IgG AlexaFluor 647‐conjugated (Abcam)

##### 2.8.11.4 Imaging

Whole slide scans of the stained sections were recorded on a Zeiss automatic microscope AxioScan Z1 with high aperture lenses, equipped with a Zeiss Axiocam 506 mono and a Hitachi 3CCD HV‐F202SCL camera and Zeiss ZEN 3.7 software.

##### 2.8.11.5 Quantification

Image analysis was done with Image Pro 10 (Media Cybernetics). At the beginning, the target area (hippocampus) was identified by drawing regions of interest (ROI) on the images. Additional ROIs exclude wrinkles, air bubbles or any other artefacts interfering with the measurement. Afterwards, signals of Iba1, GFAP and CD68 were quantitatively evaluated within the identified areas. For quantification, background correction was used if necessary, and immunoreactive objects were detected by adequate thresholding and morphological filtering (size and shape). Different object features were then quantified, among them the percentage of cumulative object area based on ROI size (immunoreactive area; this is the most comprehensive parameter indicating whether there are differences in immunoreactivity), the number of objects normalized to ROI size (object density), the mean signal intensity of identified objects (mean intensity; this indicates if there are differences in the cellular expression level of target proteins) and the size of above‐threshold objects. Once the parameters of the targeted objects were defined in a test run, the quantitative image analysis was generated automatically so that the results are operator‐independent and fully reproducible.

#### 2.8.12 Biochemistry

##### 2.8.12.1 Sample Preparation—Brain (Hippocampal Tissues)

Hippocampus of all animals (*n* = 40 samples) was homogenized 1:20 (w/v) in homogenization buffer [PBS, 1% Triton X‐100, Phosphatase Inhibitor Cocktail III (Sigma) and Protease Inhibitor Cocktail I (Calbiochem)]. Homogenates were cleared from cell debris by centrifugation at 20800 x g at 4 °C for 10 min in a tabletop centrifuge and the supernatants were collected and split into three aliquots, one of which was used for the measurement of cytokines. Protein concentrations were determined using the BCA protein assay kit from Thermo Scientific, according to the manufacturer's protocol.

##### 2.8.12.2 Measurement of Cytokine Levels

Brain (hippocampus) extracts from all animals (*n* = 40 samples) as well as terminal plasma samples (*n* = 40 samples) were diluted 1:2 and analysed for cytokines included in an inflammation panel (IL‐1*β*, IL‐6, IL‐12p70, IL‐10 and TNF*α*) with a U‐PLEX custom Cytokine Assay (K15069L‐1) from Mesoscale Discovery (MSD) and for IL‐18 with the Mouse IL‐18 DuoSet ELISA from R&D Systems (DY7625‐05). The assay was performed according to the manufacturer's instructions. Data were evaluated in comparison to the calibration curve provided in the kit and were expressed as pg/g protein for hippocampus samples or pg/mL plasma. For statistical evaluation, values below the detection limit of the assay were excluded.

All the plasma samples were analysed together, and each sample was analysed once using the U‐PLEX custom Cytokine Assay. The hippocampal protein extracts were analysed in two separate assays; during the first assay, aliquots were measured as singlets, while in the second assay, aliquots from the same samples were measured as technical duplicates. For reporting, the cytokine data of hippocampal samples from both experiments were combined and averaged.

##### 2.8.12.3 Measurement of CRF and Corticosterone

The levels of CRF and corticosterone were measured in terminal plasma samples from all mice (*n* = 40 samples), using commercially available ELISA kits according to the instructions of the manufacturer (i.e., Yanaihara Institute Inc. via BIOZOL Diagnostica cat. no. SCE‐YK131‐96 for CRF and Enzo Life Sciences cat. no. ADI‐900‐097 for corticosterone).

Prior to analysing the study samples, plasma samples from one group A (vehicle) animal and one group B (LPS, vehicle) animal were analysed in a dilution series (i.e., 1:1, 1:2, 1:4, 1:8, 1:16, 1:32, 1:64 and 1:128 for CRF and 1:10, 1:20, 1:40, 1:60 and 1:80 for corticosterone) to assess the range and dilution linearity of the assays. All study samples were analysed within the linear range of dilution (1:1 for CRF and 1:40 for corticosterone). Samples were measured as singlets. Data were evaluated in comparison to the calibration curves provided in the kit and were expressed as pg/mL plasma.

#### 2.8.13 Statistics

All raw data were analysed in GraphPad Prism 10.2.3 (GraphPad Software Inc., USA). For statistical evaluation of MSD assay data, values below the detection limit of the assay were excluded. No outlier test was performed. Normality distribution of two groups was analysed by Kolmogorov–Smirnov tests. If more than 2 groups were compared with each other, significance was calculated by one‐way or two‐way analysis of variance (ANOVA) followed by the Bonferroni post hoc test for normally distributed data. In case of non‐normally distributed data, significance was calculated by Kruskal–Wallis test followed by Dunn's multiple comparisons test. Group B (C57BL/6, LPS, vehicle) served as reference group for pairwise comparisons. Significance was defined as *p < 0.05, **p < 0.01 and ***p < 0.001.

For studies described in Section 2, detailed results of statistical analyses are provided in the figure legends.

## 3 Results

### 3.1 Effect of Nezavist on Abstinence‐Induced Escalation of Alcohol (Ethanol) Intake

#### 3.1.1 Alcohol Deprivation Effect

**FIGURE 1:**
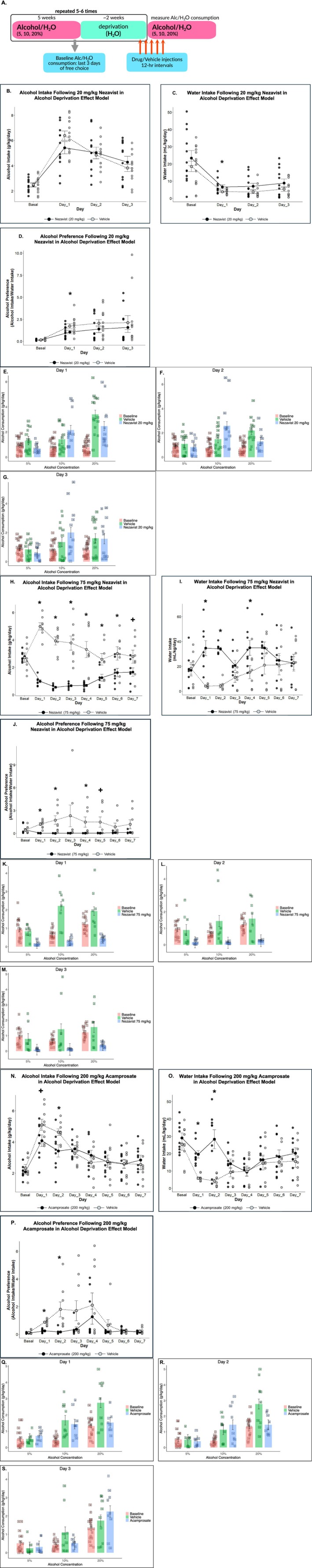
Alcohol deprivation effect model: Effect of Nezavist. (A) Experimental design of the alcohol deprivation effect model. (B–D) Alcohol Intake, water intake and alcohol preference following repeated ip dosing of 20‐mg/kg Nezavist in rat alcohol deprivation effect model. Data are presented as mean ± SEM, with solid dots indicating data from individual Nezavist‐treated rats, and clear dots indicating data from individual vehicle‐treated rats. Rats (*n* = 12–14/group) were administered vehicle or repeat doses of 20‐mg/kg Nezavist (ip). (B) Alcohol intake (g/kg/day), (C) water intake (mL/kg/day) and (D) alcohol preference (alcohol intake (g/kg)/water intake (mL/kg)) were determined prior to alcohol re‐exposure (baseline), and on Day 1, Day 2 and Day 3 after alcohol re‐exposure. Solid lines represent vehicle‐treated animals, and dashed lines represent Nezavist‐treated animals. Outliers (> 2 SDs from the mean) were removed from the analysis. Two‐way ANOVA showed a significant effect of day on alcohol intake [*F*(3, 69) = 48.197, *p* = 6.5 × 10^−17^], water intake [*F*(3, 60) = 28.28, *p* = 4.8 × 10^−7^] and alcohol preference [*F*(3, 45) = 6.573, *p* = 0.01]. *p < 0.05, ^+^ *p* = 0.12 effect of Nezavist compared to vehicle (two‐sample *t*‐test comparisons). (E–G) Consumption of different concentrations of alcohol solutions following repeated ip dosing of 20‐mg/kg Nezavist in rat alcohol deprivation model. Data are presented as mean ± SEM, with numbers in squares representing data from individual rats. Rats (*n* = 12–14 per group) were administered vehicle or repeat doses of 20‐mg/kg Nezavist (ip). The amount of each available concentration of alcohol (5%, 10% or 20%) that was consumed by vehicle or Nezavist‐treated rats at was determined at baseline (prior to alcohol re‐exposure) and on Day 1 (E), Day 2 (F) and Day 3 (G) of the alcohol deprivation effect. Outliers (> 2SDs from the mean) were treated as missing data (six of 312 observations were outliers). Three‐way ANOVA (drug treatment, day, alcohol concentration) showed a non‐significant interaction (*F* = 1.62, *p* = 0.142). Two‐way interactions between treatment group and alcohol concentration and between day and alcohol concentration were significant (*F* = 6.71, *p* = 0.0014 and *F* = 5.00, *p* = < 0.0001, respectively). On Day 1 (E), there was a suggestive interaction between drug treatment and alcohol concentration (*p* = 0.0507) with marginal significance between Nezavist and vehicle‐treated animals for consumption of 10% alcohol (*p* = 0.1514) and 20% alcohol (*p* = 0.0640). On Day 2 (F), there was a significant interaction between drug treatment and alcohol concentration (*p* = 0.0107), with a significant difference in consumption of 10% alcohol (*p* = 0.0184) and a marginal difference in consumption of 20% alcohol (*p* = 0.065) between Nezavist and alcohol‐treated animals. See Table S1 for more detail. (H–J) Alcohol intake, water intake and alcohol preference following repeated ip dosing of 75‐mg/kg Nezavist in rat alcohol deprivation effect model. Data are presented as mean ± SEM, with solid dots indicating data from individual Nezavist‐treated rats, and clear dots indicating data from individual vehicle‐treated rats. Rats (*n* = 8/group) were administered vehicle or repeat doses of 75‐mg/kg Nezavist (ip). (E) Alcohol Intake (g/kg), (F) water intake (mL/kg) and (G) alcohol preference (alcohol intake (g/kg)/water intake (mL/kg)) were determined prior to alcohol re‐exposure (Basline) and on Day 1, Day 2, Day 3, Day 4, Day 5, Day 6 and Day 7 after alcohol re‐exposure. Solid lines represent vehicle‐treated animals, and dashed lines represent Nezavist‐treated animals. Outliers (> 2 SDs from the mean) were removed from the data. Two‐way ANOVA showed a significant effect of treatment [*F*(1, 14) = 29.658, *p* = 8.62 × 10^−5^], a significant effect of day [*F*(7, 98) = 5.789, *p* = 0.001] and a significant treatment × day interaction [*F*(7, 98) = 20.809, *p* = 3.04 × 10^−8^] for alcohol intake. For water intake, two‐way ANOVA showed a significant effect of treatment [*F*(1, 11) = 13.081, *p* = 0.004, and a significant treatment × day interaction [*F*(7, 77) = 3.753, *p* = 0.02]. For alcohol preference, two‐way ANOVA showed a trend for the effect of treatment [*F*(1, 7) = 5.340, *p* = 0.054]. Post hoc pairwise *t*‐tests showed significant differences in alcohol intake between Nezavist and vehicle for Days 1–6 (*p = 1.15 × 10^−7^, 5.93 × 10^−6^, 4.65 × 10^−4^, 0.0029, 0.0192 and 0.0336) and for water intake on Days 1, 2 and 4 (*p = 1.5 × 10^−3^, 1.22 × 10^−8^, 0.028). For alcohol preference, two‐sample *t*‐tests showed differences between Nezavist and vehicle on Days 1, 2, 4 (*p < 0.05) and 5 (+*p* = 0.14). (K–M) Consumption of different concentrations of alcohol solutions following repeated ip dosing of 75‐mg/kg Nezavist in rat alcohol deprivation model. Data are presented as mean ± SEM, with numbers in squares representing data from individual rats. Rats (*n* = 8 per group) were administered vehicle or repeat doses of 75‐mg/kg Nezavist (ip). The amount of each available concentration of alcohol (5%, 10% or 20%) that was consumed by vehicle‐ or Nezavist‐treated rats was determined at baseline (prior to alcohol re‐exposure) and on Day 1 (K), Day 2 (L) and Day 3 (M) of the alcohol deprivation effect. Outliers (15 values > 2SDs from the mean) were treated as missing data. Three‐way ANOVA (drug treatment, day and alcohol concentration) showed a non‐significant interaction (*F* = 0.73, *p* = 0.744), while two‐way interaction between treatment group and alcohol concentration was significant (*F* = 3.15, *p* = 0.044). On Day 1, Nezavist‐treated animals reduced consumption of 5%, 10% and 20% alcohol compared to vehicle‐treated animals (*p* = 6.08e‐02, 1.16e‐06 and 6.64e‐05, respectively). On Day 2, Nezavist‐treated animals reduced consumption of all three concentrations of alcohol, compared to vehicle‐treated animals, with a significant effect on 10% and 20% alcohol (*p* = 0.008 and 0.01, respectively). On Day 3, Nezavist‐treated animals again reduced consumption of all three concentrations of alcohol, with significant effects on 10% and 20% alcohol (*p* = 0.014 and 0.022, respectively). (N–P) Alcohol intake, water intake and alcohol preference following repeated ip dosing of 200‐mg/kg Acamprosate in rat alcohol deprivation effect model. Data are presented as mean ± SEM, with solid dots indicating data from individual Acamprosate‐treated rats and clear dots indicating data from individual vehicle‐treated rats. Rats (*n* = 9/group) were administered vehicle or repeat doses of 200‐mg/kg Acamprosate. (H) Alcohol intake (g/kg), (I) water intake (mL/kg) and (J) alcohol preference (alcohol intake (g/kg)/water intake (mL/kg)) were determined prior to alcohol re‐exposure (basline) and on Day 1, Day 2, Day 3, Day 4, Day 5, Day 6 and Day 7 after alcohol re‐exposure. Solid lines represent vehicle‐treated animals, and dashed lines represent Acamprosate‐treated animals. Outliers (> 2 SDs from the mean) were removed from the data. For alcohol intake, two‐way ANOVA showed a significant effect of day [*F*(7, 98) = 21.254, *p* = 3.58 × 10^−17^] and a significant treatment × day interaction [*F*(7, 98) = 3.08, *p* = 0.006]. For water intake, two‐way ANOVA showed a significant treatment effect [*F*(1, 16) = 5.663, *p* = 0.03], a significant day effect [*F*(7, 112) = 9.502, *p* = 6.28 × 10^−5^ and a significant treatment × day interaction [*F*(7, 112) = 5.437, *p* = 0.003]. Post hoc pairwise *t*‐tests showed differences between Acamprosate and vehicle for alcohol intake and water intake on Days 1 and 2 (*p < 0.02, +*p* < 0.06). Two‐sample *t*‐test showed differences (*p < 0.05) between Acamprosate and vehicle for alcohol preference on Days 1 and 2. (Q–S) Consumption of different concentrations of alcohol solutions following repeated ip dosing of 200‐mg/kg Acamprosate in rat alcohol deprivation model. Data are presented as mean ± SEM, with numbers in squares representing data from individual rats. Rats (*n* = 9 per group) were administered vehicle or repeat doses of 200‐mg/kg Acamprosate (ip). The amount of each available concentration of alcohol (5%, 10% or 20%) that was consumed by vehicle‐ or Nezavist‐treated rats was determined at baseline (prior to alcohol re‐exposure) and on Day 1 (Q), Day 2 (R) and Day 3 (S) of the alcohol deprivation effect. Outliers (18 values > 2SDs from the mean) were treated as missing data. Three‐way ANOVA (drug treatment, day, alcohol concentration) showed a significant interaction (*F* = 2.14, *p* = 0.0096), while the two‐way interaction between treatment group and alcohol concentration was suggestive (*F* = 2.97, *p* = 0.053). Because of the three‐way interaction, the data were stratified by day, and the interaction between treatment group and alcohol concentration was examined. On Day1, Acamprosate‐treated animals reduced consumption of 10% and 20% alcohol, compared to vehicle‐treated animals, with a significant effect on 20% alcohol (*p* = 0.0076). On Day 2, Acamprosate‐treated animals reduced consumption of 5% and 20% alcohol, compared to vehicle‐treated animals, with a significant effect on 20% alcohol (*p* = 0.005). On Day 3, Acamprosate‐treated animals reduced consumption of 10% alcohol, compared to vehicle‐treated animals, but this effect was not significant.

The ‘alcohol deprivation effect’ model with repeated deprivation phases as described by Spanagel et al. [57, 58] involves long term (10 months) consumption of alcohol (ethanol) by rats with intermittent periods of abstinence. When animals regain access to alcohol following the deprivation period, there is a statistically significant increase in alcohol consumption for several days after alcohol reintroduction. Figure 1B–D from our studies shows results of treatment with repeated [5] doses of 20‐mg/kg Nezavist. Total alcohol intake was significantly increased over basal levels on the first day and for the two succeeding days after alcohol re‐exposure in both the vehicle and Nezavist‐treated groups (ANOVA, *p* = 6.5 × 10^−17^), but the escalation of total alcohol consumption by the vehicle‐treated group during the first day after reintroduction of alcohol was modestly blunted in the group that received the Nezavist treatment (two‐sample *t*‐test, *p* = 0.12, Figure 1B). In both Nezavist and vehicle‐treated animals, the consumption of water was significantly *decreased* on the first and subsequent days, in conjunction with the increased consumption of the alcohol solution after reintroduction of alcohol availability (ANOVA, *p* = 4.8 × 10^−7^, Figure 1C). Nezavist administration significantly reduced this *decrease* in water consumption on Day 1 (*p* < 0.05). Thus, when alcohol preference was calculated (amount of alcohol consumed/quantity of water consumed per day) for each animal, there was a significant increase in preference over the 3‐day period in both groups (ANOVA, *p* = 0.01), but the administration of Nezavist (20 mg/kg × 5) produced a significant diminution of alcohol preference on the first day of post‐abstinence alcohol consumption, compared to vehicle‐treated rats (Figure 1D, *p* < 0.05). There was no significant effect of the Nezavist treatment on locomotor activity or body weight (data not shown).

When the quantity of each of the concentrations of alcohol (5%, 10% and 20%) consumed by the Nezavist (20 mg/kg × 5)‐treated and vehicle‐treated rats at baseline and during the initial 3 days after the deprivation period was examined individually (Figure 1E–G), a three‐way ANOVA (drug treatment, day and alcohol concentration) indicated that the three‐way interaction among these factors was not significant (*p* = 0.142). However, both the two‐way interaction between drug treatment and alcohol concentration (*p* = 0.001) and the two‐way interaction between day and alcohol concentration (*p* < 0.0001) were significant. Therefore, for further analyses, the data were stratified by day to help with interpretation of these interactions. As expected, at baseline, prior to alcohol deprivation and administration of Nezavist, there was no main effect of drug (Nezavist) treatment (*p* = 0.99) and no interaction of drug treatment and alcohol concentration (*p* = 0.31). Interestingly, there was no main effect of alcohol concentration at baseline (*p* = 0.74) (Figure 1E–G). Among the 3 days after administration of Nezavist, there was a significant interaction effect between Nezavist treatment and alcohol concentration on Day 2 (*p* = 0.012) and a suggestive interaction on Day 1 (*p* = 0.051) (Table S1), indicating that the effect of Nezavist on alcohol consumption differed from vehicle based on alcohol concentration. On Day 1, vehicle‐treated animals consumed the highest amount of alcohol by consumption of the 20% solution (3.41 g of alcohol/kg body weight for the 20% solution, 1.43 g/kg body weight for the 10% solution and less for the 5% solution). Treatment with Nezavist (20 mg/kg/dose) reduced the amount of alcohol ingested by drinking the 20% solution (2.16‐g/kg body weight, marginal significance, *p* = 0.064), and there was an increase in the quantity of alcohol consumed by drinking the 10% solution (also 2.16‐g/kg body weight for the 10% solution, *p* = 0.151 compared with vehicle‐treated animals) (Figure 1E). On Day 2, although the increase in consumption of the 10% solution by the Nezavist‐treated animals remained relatively constant, due to reduced variance, there was a significant difference between vehicle and Nezavist‐treated animals (*p* = 0.018, Figure 1F). Furthermore, as on Day 1, the increase in consumption of the 10% alcohol solution was compensated by a trend toward a decrease in the consumption of the 20% alcohol concentration (Day 2, *p* = 0.065). It should be noted that the full treatment with Nezavist was not completed until Day 2. On Day 3, the values for consumption of the 10% and 20% alcohol solutions by Nezavist‐treated animals were similar to the levels noted on Day 2, but with no significant differences compared to vehicle‐treated animals. Overall, on Day 1, the changes in consumption of the 10% and 20% solutions resulted in a diminution of the total alcohol consumed (Figure 1B).

Increasing the dose of Nezavist to 75‐mg/kg body weight ip, given five times over the 2.5‐day period, substantially increased the effects on alcohol and water consumption over a 7‐day period subsequent to the reintroduction of alcohol availability after forced abstinence (Figure 1H–J). The effect of alcohol deprivation to increase alcohol intake was again readily evident in the vehicle group. However, a dose of 75‐mg/kg body weight of Nezavist, with the first dose being given on the day prior to alcohol availability after the 14‐day deprivation period, and on two subsequent days, generated a significant *decrease* in the total amount of alcohol consumed on the first day of alcohol reintroduction compared to baseline alcohol consumption prior to the 14‐day deprivation period (ANOVA, *p* = 3.04 × 10^−9^). A significant difference (*p* < 0.05) in total alcohol intake persisted between the vehicle‐treated and the Nezavist‐treated groups for 6 days after reintroduction of alcohol following the deprivation period. In the vehicle‐treated group, alcohol consumption was increased and water consumption was decreased in conjunction with the increased alcohol intake. In contrast, alcohol intake was *decreased* by administration of Nezavist, and water intake was significantly *increased* over baseline in the group of animals treated with Nezavist, compared to the vehicle‐treated group (ANOVA, *p* = 0.02). The presentation of results as a preference ratio demonstrates that alcohol preference was close to zero in the Nezavist‐treated group for the 7 days following reintroduction of alcohol, while the alcohol preference ratio remained above 1 for 5 days after the reintroduction of alcohol in the vehicle‐treated group. Overall, there was a significant effect of Nezavist (75‐mg/kg body weight) on alcohol preference during the days (Days 1 and 2) when Nezavist was being administered (lower alcohol intake/higher water intake, *p* < 0.05), and this effect remained evident for at least 2 days after terminating Nezavist administration (*p* < 0.05). The Nezavist‐treated animals displayed a decrease in total fluid intake over the course of the experiment, but the animals consumed ~35 mL/kg/day of water on days when the effect of Nezavist was evident, suggesting that the animals were not dehydrated (https://policies.unc.edu/TDClient/2833/Portal/KB/ArticleDet?ID=132199) but are mainly reducing alcohol‐containing fluid consumption.

When the quantity of each of the concentrations of alcohol consumed by the Nezavist (75 mg/kg × 5)‐treated and vehicle‐treated rats at baseline and during the initial 3 days after the deprivation period was examined individually (Figure 1K,L), the three‐way ANOVA was not significant (*p* = 0.142), but both the two‐way interaction between drug treatment and alcohol concentration (*p* = 0.044) and the two‐way interaction between day and treatment (*p* = 0.0002) were significant. Therefore, for further analyses, the data were stratified by day to simplify interpretation of these interactions. On Day 1 of the alcohol deprivation period, Nezavist treatment produced a significant diminution of all of the concentrations of alcohol, compared to vehicle‐treated animals (5%, *p* = 6.08e‐02; 10%, *p* = 1.16e‐06; 20%, *p* = 6.64e‐05;Figure 1K). During Day 2, there was a significant diminution of consumption of the 10% (*p* = 0.008) and 20% (*p* = 0.01) alcohol solutions by the Nezavist‐treated animals, but the consumption of the 5% solution was no longer significantly different from that of the vehicle‐treated animals (Figure 1L). The same pattern was noted on Day 3 (10% solution, *p* = 0.014; 20% solution *p* = 0.023; Figure 1M). On Day 4, only the consumption of the 10% solution was reduced in the Nezavist‐treated animals compared to vehicle‐treated animals (*p* = 0.005). From Day 5 onward, alcohol consumption by the Nezavist‐treated animals was reduced at all concentrations, but there were no statistically significant effects. However, the total overall consumption of alcohol was significantly lower on Days 5 and 6, and marginally lower on Day 7 in the Nezavist‐treated rats compared to vehicle‐treated rats (Figure 1H).

There was a minimal loss of body weight in the Nezavist‐treated animals (vehicle‐treated rats gained 0.9% of their starting body weight, while Nezavist‐treated rats lost 1.3% of their starting body weight over the course of the experiment). Locomotor activity was decreased in the Nezavist‐treated animals for the first 2 days of re‐exposure to alcohol (ANOVA, *p* < 0.05) (Figure S1) but returned to the level of the vehicle‐treated animals by the third day of re‐exposure, while alcohol intake and preference remained significantly decreased. This finding suggests that the decrease in locomotor activity did not cause the decrease in alcohol intake and could not account for the increased water intake. However, the decrease in locomotor activity may have contributed to decreased food intake, reflected in the small decrease in body weight of the Nezavist‐treated animals.

Acamprosate (200‐mg/kg body weight × 5 ip) (used here as a comparator drug to Nezavist) produced a significant decrease in total alcohol consumption (*p* < 0.05) during the second day after the reintroduction of the alcohol solutions, compared to the vehicle‐treated rats (Figure 1N). This was primarily due to a diminution of consumption of the 20% alcohol solution (*p* = 0.0047; Figure 1R). The overall alcohol consumption was no longer significantly reduced by the third day of re‐exposure, when there were no significant differences in the consumption of any of the individual concentrations of alcohol (Figure 1S). In addition, the alcohol consumption of the Acamprosate‐treated rats did not fall below the alcohol consumption levels demonstrated by rats during the baseline consumption period prior to deprivation. Water consumption levels were higher in the Acamprosate‐treated animals compared to vehicle‐treated animals on the first 2 days of alcohol re‐exposure (*p* < 0.05), resulting in reduced alcohol preference on those days (*p* < 0.05) (Figure 1O,P). Alcohol intake and preference in the Acamprosate‐treated rats returned to the baseline level by Day 3 of alcohol re‐exposure. There were no significant differences in body weight or locomotor activity between the vehicle‐ and Acamprosate‐treated animals (data not shown).

#### 3.1.2 Operant Responding for Alcohol (Ethanol)

**FIGURE 2:**
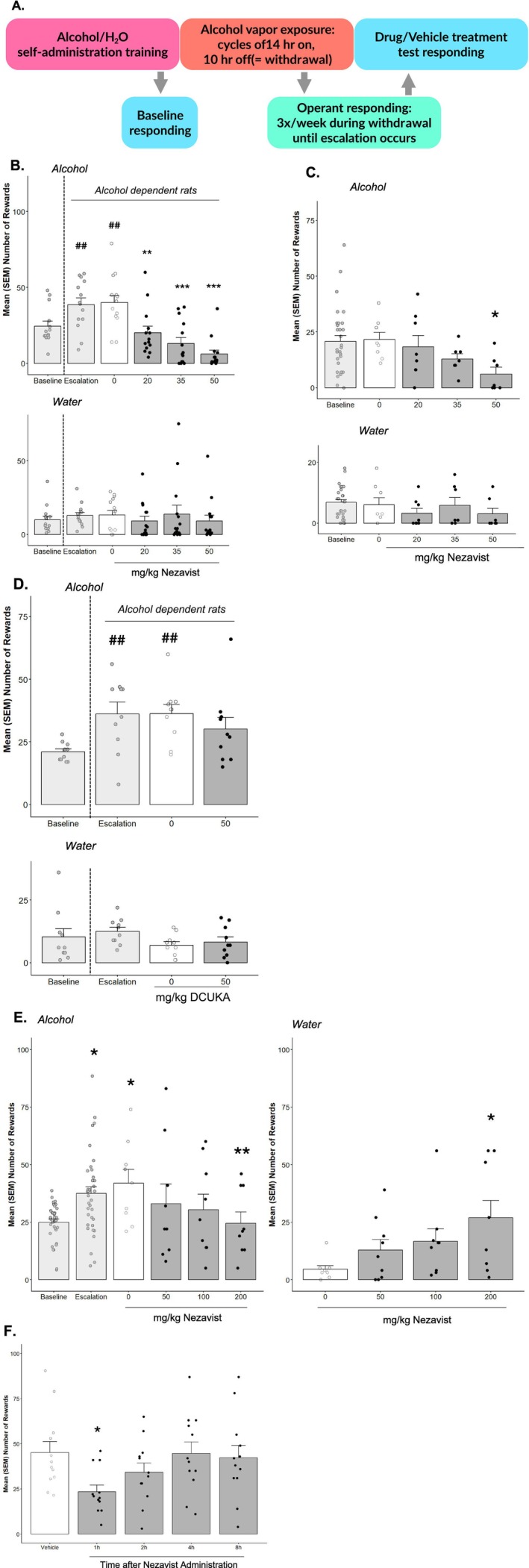
Operant responding for alcohol and water. (A) Experimental design of operant responding model. (B) Effect of ip Nezavist on alcohol and water operant self‐administration by alcohol‐dependent rats; 14 rats were used for this experiment. Drug treatment used a within‐subject Latin square design. Values represent the mean ± SEM of the number of rewards at the alcohol and the water lever. BSL = baseline pre‐vapour. ESC = baseline post escalation. After chronic vapour exposure and withdrawal, animals showed escalation of responding for alcohol (*t* = 3.384, df = 14, ^##^ *p* < 0.01 vs. BSL); One‐way ANOVA showed a significant effect of treatment: *F*(3, 13) = 16.98, *p* < 0.001. Newman–Keuls post hoc tests showed that all doses of Nezavist reduced operant responding (**p < 0.01 and ***p < 0.001 vs. Dose 0). Nezavist treatment did not modify water self‐administration [*F*(3, 13) = 0.50; *p* = NS]. (C) Effect of ip Nezavist on alcohol and water operant self‐administration by nondependent rats. Values represent the mean ± SEM of the number of rewards (lever presses) at the alcohol and the water lever. A total of 30 rats were used in this experiment. After training, rats were divided into four treatment groups (7–8/group). One‐way ANOVA showed a significant effect of treatment [*F*(3, 26) = 4.747, *p* < 0.05]. Neuman–Keuls post hoc test showed that 50 mg/kg of Nezavist reduced alcohol intake, *p < 0.05 vs. Dose 0. BSL = baseline pre‐treatment. (D) Effect of ip DCUKA on alcohol and water operant self‐administration by alcohol‐dependent rats. Values represent the mean ± SEM of the number of rewards (lever presses) for the alcohol and the water levers. A total of 10 rats were used in this experiment and were treated according to a Latin square design. BSL = baseline pre‐vapour. ESC = baseline post escalation. *t* = 3.626, df = 9, ## *p* < 0.01 vs. BSL. (E) Effect of orally administered Nezavist on alcohol and water operant self‐administration by alcohol‐dependent rats. Values represent mean ± SEM number of rewards, *n* = 9 rats/group. BSL = baseline pre‐vapour; ESC = baseline post escalation. Alcohol rewards: one‐way ANOVA including BSL and ESC groups showed a significant effect of treatment: [*F*(5, 102) = 3.299; *p* < 0.01]. Newman–Keuls post hoc test showed a significant escalation of intake in the ESC and dose 0 groups (*p < 0.01 and *p < 0.05, respectively) when compared to the BSL group. When the four Nezavist doses were analysed with four separate one‐way ANOVAs, the results showed that only the treatment with the 200‐mg/kg dose of Nezavist significantly reduced alcohol intake [*F*(2, 8) = 6.02; *p* < 0.05]. The Newman–Keuls post hoc test showed significantly reduced operant responding for alcohol by these animals (** *p* < 0.05). Water rewards: Water intake was affected by the treatment with Nezavist [*F*(3, 32) = 3.142; *p* < 0.05]. Newman–Keuls post hoc test showed a significant increase in water consumption in animals treated with 200‐mg/kg Nezavist, compared with Dose 0 (*p < 0.05). (F) Time course of effect of orally administered Nezavist (200 mg/kg) on operant alcohol self‐administration by alcohol‐dependent rats. Values represent the mean ± SEM number of rewards at the alcohol lever (*n* = 12/group). One‐way ANOVA showed a significant effect of treatment [F (4, 55) = 2.593, *p* < 0.05]. Nezavist was effective when administered 1 h prior to testing (*p < 0.05, Neuman–Keuls test).

The method described in [37, 38, 39] was used to evaluate the effect of Nezavist on the increase in alcohol intake that occurs in alcohol‐dependent animals during withdrawal from chronic alcohol exposure. The escalation of alcohol intake (operant responding) after alcohol withdrawal in dependent rats is considered to be a model of negative reinforcement alcohol seeking [6]. In the first experiment (Figure 2B), Nezavist was administered ip to dependent rats 30 min prior to the testing session. Nezavist significantly reduced responding for alcohol in a dose‐dependent manner, with no change in responding for water (ANOVA, *p* < 0.001). The effect of Nezavist in rats trained to respond for alcohol but *not* made dependent on alcohol (no exposure to alcohol vapour) was also determined. Nezavist, administered ip, was less potent in the nondependent rats (Figure 2C), with a significant effect only at a dose of 50 mg/kg (ANOVA, *p* < 0.05). Since Nezavist pharmacokinetic experiments, described below, showed very low or undetectable levels of Nezavist in the circulation after ip administration but higher levels of the major metabolite, DCUKA, the effect of DCUKA on the escalation of alcohol responding, was also assessed (Figure 2D). DCUKA (50 mg/kg), administered ip at a dose equivalent to the highest dose of Nezavist tested, did not affect responding for alcohol or water in the alcohol‐dependent rats (*t*‐test, *p* > 0.05).

The effect of orally administered Nezavist on operant responding by the alcohol‐dependent rats was also evaluated (Figure 2E). Nezavist was delivered by oral gavage 1 h prior to testing and was effective in reducing alcohol intake by the alcohol‐dependent rats. However, compared to Nezavist administration by the ip route, a higher oral dose (200 mg/kg) was needed to produce a significant effect (ANOVA, *p* < 0.05). With the oral administration, there was a significant increase in water consumption in conjunction with the diminished lever pressing for alcohol (ANOVA, *p* < 0.05). A follow‐up experiment with orally administered Nezavist demonstrated a peak suppressive effect of Nezavist on alcohol responding at 1 h after administration (ANOVA, *p* < 0.05), and the effect was no longer evident by 4 h after administration (Figure 2F).

Overall, the results in the operant responding model, using alcohol‐dependent rats, provide a similar picture as those seen with the alcohol deprivation effect, i.e., Nezavist demonstrates a dose‐dependent effect in reducing alcohol intake in dependent animals (‘negative reinforcement‐induced alcohol relapse’). It is also interesting that the threshold effective dose of Nezavist in either model was similar when Nezavist was administered ip. These results also show that Nezavist is less potent in nondependent animals, which may be an important feature for the use of Nezavist in humans.

### 3.2 General Phenotyping and Other Alcohol‐Related Behaviours

#### 3.2.1 Locomotor Activity and Incoordination (Rotarod)

**FIGURE 3:**
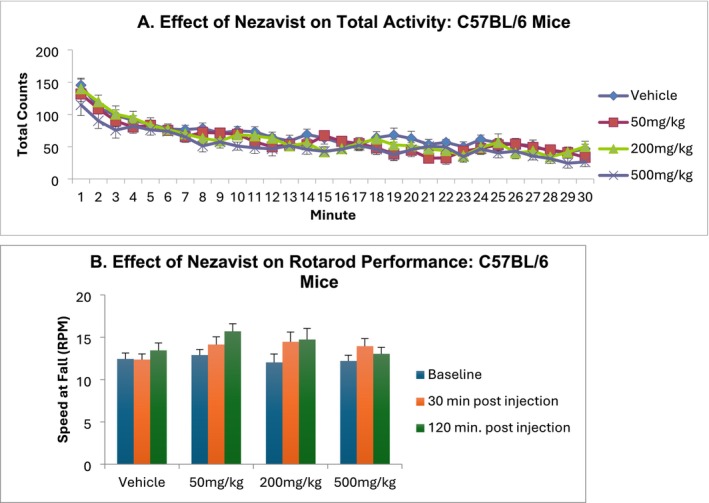
Effect of ip nezavist on mouse locomotor activity and rotarod performance. (A) Total activity: Values are mean ± SEM (*n* = 10/group) of activities measured, including ambulation and rearing, over time, for each dose group. ANOVA showed no significant effect of dose [*F*(3, 36) = 1.326, *p* = 0.28]. There was an effect of time: [*F*(3, 29) = 45.204, *p* < 0.0001] but no significant time × dose interaction [*F*(3, 87) = 0.987, *p* = 0.515]. (B) Rotarod performance. Values are mean ± SEM (*n* = 10/group) of speed (rpm) at which mice fell off the rotarod. ANOVA showed no significant effect of dose: [*F*(3, 36) = 0.711, *p* = 0.552]. There was a significant effect of time: [*F*(2, 3) = 13.95, *p* < 0.0001) and a trend toward a dose × time interaction: [*F*(3, 6) = 2.192, *p* = 0.0535]. However, the animals given doses of 50 or 200 mg/kg of Nezavist *improved* their performance by falling off at a higher speed at 30 and 20‐min posttreatment, compared to speed after vehicle treatment.

Results for locomotor activity in mice are shown in Figure 3A. The effect of Nezavist on ambulation, center activity, rearing activity and total activity was assessed in mice given 50‐, 200‐ or 500‐mg/kg Nezavist ip. There were no apparent effects of Nezavist on total ambulation or total rearing activity, although total center activity was significantly (*p* < 0.05) reduced in mice treated with 500‐mg/kg Nezavist, compared to vehicle‐treated mice. Total activity, i.e., a combination of ambulation and rearing activity, was not affected by any dose of Nezavist (ANOVA, *p* = 0.21).

Results for incoordination in mice are shown in Figure 3B. The speed of the rotarod was recorded when the animal fell at 0 min (baseline, predose), 30 min postinjection and 120 min postinjection. Three doses of Nezavist were administered via ip injection to mice: 50, 200 or 500 mg/kg. Nezavist did not produce impairment in the rotarod test at any dose.

#### 3.2.2 Elevated Plus Maze (Anxiety)

**FIGURE 4:**
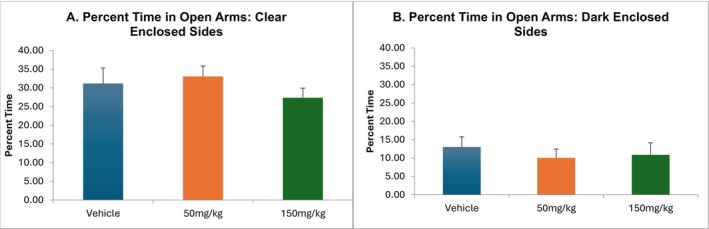
Effect of ip Nezavist on anxiety‐like behaviour in the elevated plus maze in mice. Values are mean ± SEM percent of time spent in open arms of two different plus mazes (*n* = 10/group). Nezavist had no significant effect on time in the open arms either in the clear enclosed sides maze (A), ANOVA: [*F*(2, 27) = 0.803, *p* = 0.458], or in the dark enclosed sides maze (B), ANOVA: [*F*(2, 27) = 0.29, *p* = 0.751]. Nezavist did reduce *total* arm entries, a measure of activity, in the clear enclosed sides maze only, ANOVA: [*F*(2, 27) = 8.86, *p* = 0.001], Fishers PLSD for arm entries, *p* < 0.05 vehicle and 50‐mg/kg dose vs. 150‐mg/kg dose.

In the elevated plus maze test to assess anxiety‐like behaviour, Nezavist did not affect the percent open arm time for either of the two mazes (Figure 4, ANOVA, *p* = 0.458 for clear enclosed sides; *p* = 0.751 for dark enclosed sides). More time on open arms would be indicative of decreased anxiety‐like behaviour. There was an effect of Nezavist on *total* arm entries (a measure of activity) for the Clear Enclosed Sides maze (ANOVA, *p* < 0.001), with the 150‐mg/kg dose significantly decreasing this measure (*p* < 0.05). However, Nezavist did not affect total arm entries in the other (Dark Enclosed Sides) maze. Overall, the results suggest that Nezavist has no significant effect on anxiety‐like behaviour measured in this test in mice. While Nezavist produced a small decrease in activity levels at the higher dose, this was not consistent across the two mazes.

Nezavist was also tested for anxiolytic and anxiogenic effects in seven other tests: startle response, prepulse inhibition, light/dark transfer test, sociability test, marble burying test, shock‐induced freezing and stress‐induced hyperthermia. There were no significant effects of up to 150‐mg/kg Nezavist, administered ip, in these tests (data not shown).

#### 3.2.3 Forced Swim Test (An Acute Stress Coping Strategy)

**FIGURE 5:**
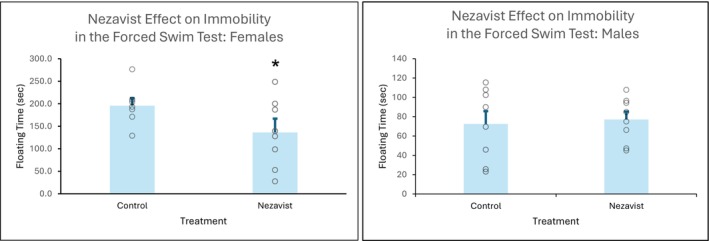
Effect of Nezavist in the Porsolt (Forced Swim) test as a measure of stress coping activity. Effect of Nezavist (50 mg/kg, ip) on immobility in the Porsolt forced swim test. Male or female rats (*n* = 8) were treated with Nezavist or vehicle 90 min prior to the swim test. Values are mean ± SEM of time (sec) spent floating (immobility) during the 5‐min test. In females, immobility was significantly decreased by Nezavist (*p = 0.04, *t*‐test). Nezavist did not affect immobility in the male rats.

In our study, ‘floating time’ was measured, as it captures limb movements, even when the center point of the animal is considered to be immobile. Figure 5 shows that female rats treated with 50‐mg/kg Nezavist ip, 90 min before testing, showed significantly decreased floating time (immobility) compared to vehicle‐treated female animals (*t*‐test, *p* = 0.04). There was no significant difference between vehicle‐treated and Nezavist‐treated male rats in this test.

#### 3.2.4 Interaction with Ethanol (Alcohol): Sedation (Loss of Righting Reflex) and Incoordination (Rotarod)

**FIGURE 6:**
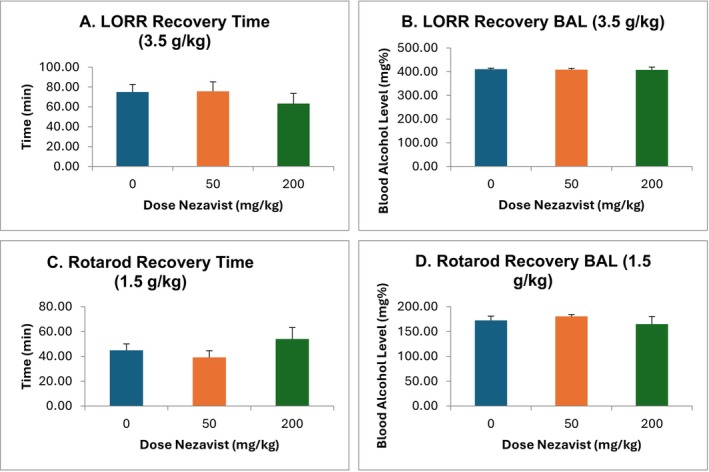
Interaction of ip Nezavist with alcohol on rotarod performance and sedation in male C57BL/6 mice. Interaction of Nezavist (50 or 200 mg/kg ip) with alcohol (1.5 g/kg or 3.5 g/kg ip) on sedation, as measured by loss of righting reflex (LORR), and rotarod performance in male C57BL/6 mice (*n* = 6/group). Values are mean ± SEM time to regain righting reflex (A) or time to regain balance on the rotarod (C); and corresponding blood alcohol levels (BAL) at regain of function (B and D). Nezavist did not affect the responses to alcohol. ANOVAS: LORR recovery [*F*(2, 15) = 0.560, *p* = 0.579]; LORR BAL, [*F*(2, 15) = 0.036, *p* = 0.965]; Rotarod recovery, [*F*(2, 14) = 1.246, *p* = 0.318]; Rotarod BAL, [*F*(2, 14) = 0.614, *p* = 0.56].

To assess the interaction of Nezavist and alcohol on sedation (loss of righting reflex), mice were injected ip with vehicle or Nezavist (50 or 200 mg/kg) 30 min prior to ip injection with 3.5 g/kg of alcohol. Figure 6A,B shows that neither dose of Nezavist affected the time for the mice to regain the righting reflex (A) or the blood alcohol level at which the righting reflex was regained (B).

To assess the interaction of Nezavist and a lower dose of alcohol on incoordination, mice were injected ip with vehicle, 50‐ or 200‐mg/kg Nezavist and 30 min later were injected ip with 1.5‐g/kg alcohol. Mice were then tested for ability to maintain balance on the rotarod for 30 s at 7 rpm. Figure 6C,D shows that Nezavist did not affect the time for the mice to regain performance on the rotarod after alcohol treatment (C) and did not affect the blood alcohol level at the time when balance was regained (D). These results indicated that Nezavist did not affect alcohol metabolism.

#### 3.2.5 Conditioned Place Preference (Abuse Potential)

**FIGURE 7:**
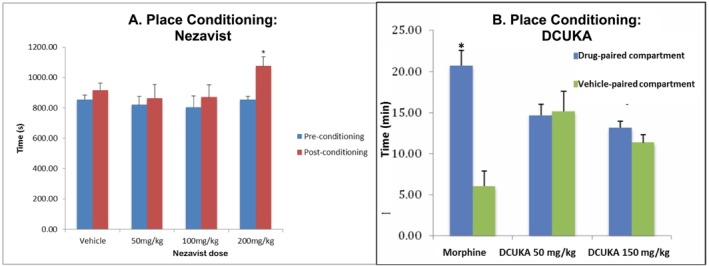
Addictive potential of Nezavist: Conditioned place preference. Values are mean ± SEM. (A) Time spent in the compartment paired with Nezavist on the test day (‘postconditioning’; *n* = 10 C57BL/6 male mice/group). Nezavist (200 mg/kg) given ip significantly increased time spent in the paired compartment [ANOVA for 200 mg/kg pre vs. post, *F*(1, 9) = 12.87, *p* = 0.0059]. (B) Time spent in the drug‐paired or vehicle‐paired compartment on the test day (*n* = 10 CF‐1 male mice/group). Morphine (10 mg/kg) significantly increased time spent in the drug‐paired compartment (*t*‐test, *p < 0.001 compared to vehicle‐paired compartment). DCUKA‐treated mice (50 or 150 mg/kg) did not display significant place preference (*t*‐test, *p* > 0.05, compared to vehicle‐paired compartment).

Figure 7A shows that mice treated with Nezavist (200 mg/kg ip) showed a small but significant increase in conditioned place preference (20% increased time spent in the drug‐paired environment, ANOVA, *p* = 0.0059), while mice treated with the lower doses of Nezavist (50 or 100 mg/kg) did not. Figure 7B shows that morphine, the positive control, displayed a significant place preference (*t*‐test, *p* < 0.001 vs. vehicle‐paired compartment), and the Nezavist metabolite, DCUKA (50 or 150 mg/kg), did not. The results indicate that Nezavist and DCUKA have little to no addictive potential, consistent with low to no detectable brain levels of drug after ip or oral administration (see below).

### 3.3 Pharmacokinetic Studies of Nezavist and the Major Metabolite (DCUKA) in Rats

#### 3.3.1 Blood and Brain Levels of Nezavist and DCUKA after ip Administration

**FIGURE 8:**
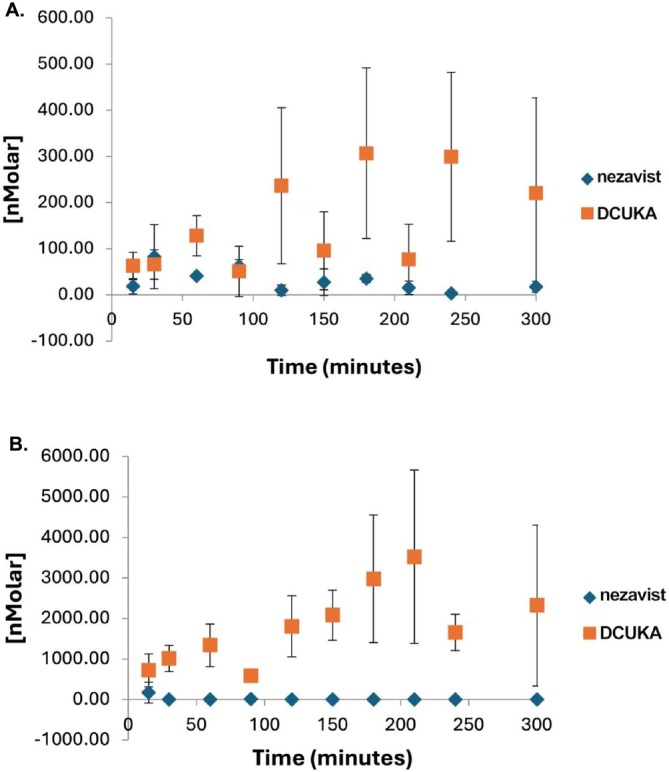
Pharmacokinetics of Nezavist. (A) Blood levels were measured in Sprague–Dawley rats following a single ip dose of 50 mg/kg of Nezavist. Values represent mean ± SD (*n* = 3 animals/time point). (B) Blood levels of Nezavist and DCUKA after the last of three consecutive ip injections of 50‐mg/kg Nezavist (total 150 mg/kg) spaced 2 h apart. Values represent mean ± SD (*n* = 3 animals/time point).

Levels of Nezavist and the major metabolite, DCUKA, in whole blood were quantified following administration (ip) of Nezavist at doses of 50 mg/kg, 3 × 50 mg/kg (one dose every 2 h), or 400 mg/kg to male Sprague Dawley rats. The rationale for administering multiple and high doses of Nezavist was to determine if blood and brain levels of Nezavist could be increased by accumulation after multiple doses of Nezavist were administered. For all doses of Nezavist that were administered, blood levels of Nezavist were low to undetectable. Blood levels of *DCUKA* were variable, but measurable (nM‐μM) after all Nezavist doses. DCUKA levels were lowest in the animals receiving a single 50‐mg/kg dose of Nezavist (Figure 8A) and increased in those receiving 3 × 50 mg/kg over a 4‐h period (Figure 8B). Blood levels of DCUKA were no higher after a single dose of 400 mg/kg than after 3 × 50 mg/kg doses (data not shown).

In Sprague Dawley rats treated with 50 mg/kg or 3 × 50 mg/kg Nezavist (ip), brain levels of Nezavist, if detectable, were in the nM range, were variable and most often were below the level of quantification (BQL) (Table 1). Brain levels of DCUKA were low (in the nM range) but increased with increasing doses of Nezavist.

**TABLE 1:** Brain levels of Nezavist and DCUKA after ip Nezavist administration.

Nezavist	Brain collection time	Nezavist	DCUKA
Dose	(Hrs. postdose)	(nM)	(nM)
1 × 50 mg/kg	1	6.3 ± 6.3	10.0 ± 10.0
	2	81.4 ± 71.0	47.4 ± 12.8
	3	13.4 ± 13.4	27.3 ± 1.7
3 × 50 mg/kg	1	27.7 ± 7.3	50.4 ± 11.0
	2	0	77.4 ± 3.0
	3	15.4 ± 7.7	69.1 ± 34.6

*Note:* Values represent mean ± SEM (*n* = 3). In some instances, values were < LLOQ (2.5 ng/g).

#### 3.3.2 Blood, Brain and Liver Levels of Nezavist and DCUKA After Oral Administration of Nezavist.

Concentrations of Nezavist and DCUKA were measured in whole blood, liver and brain after oral administration of 50‐ or 150‐mg/kg Nezavist to male Sprague Dawley rats (Table 2). The levels of Nezavist in blood after either dose, and at all time points tested up to 120 min, were below the limit of quantification (BQL, < 1 ng/mL = < 2 nM). Nezavist and DCUKA were also measured in liver and brain tissue. Nezavist was detected in rat liver, reaching 41 and 57 nM at 30 min after administration of 50 or 150 mg/kg, respectively (Table 3). DCUKA levels were much higher than Nezavist levels in liver, reaching approximately 7 μM at 1 h after either dose of Nezavist. When brain levels of Nezavist were measured, Nezavist was BQL (< 7.5 ng/g = 16 nM) at all time points tested. DCUKA levels in the brain were low, compared to those seen in blood or liver; 33 nM DCUKA was measured in rat brain at 60 min after administration of 50 mg/kg of Nezavist, and about 40 nM at 60 min after administration of 150 mg/kg of Nezavist, but in several samples at other time points, levels were BQL (Table 4).

**TABLE 2:** Blood Levels of Nezavist and DCUKA after oral administration of Nezavist.

Nezavist (nM)				
Nezavist dose	30 min	60 min	90 min	120 min
50 mg/kg	BQL	BQL	BQL	BQL
150 mg/kg	BQL	BQL	BQL	BQL
250 mg/kg SDD	BQL	BQL	BQL^+^	BQL*
DCUKA				
50 mg/kg	207.5 ± 10.1 nM	194.6 ± 16.2 nM	197.6 ± 21.4 nM	173.6 ± 17.5 nM
150 mg/kg	212.5 ± 23.3 nM	248.4 ± 32.3 nM	174.7 ± 11.8 nM	163.5 ± 20.9 nM
250 mg/kg (SDD)	25.4 ± 2.5 μM	67.2 ± 11.3 μM	67 ± 16.9 μM	66.3 ± 18.3 μM

*Note:* 50 and 150 mg/kg: Values represent mean ± SEM (30 min, *n* = 9; 60 min, *n* = 6; 90 and 120 min, *n* = 3). Nezavist BQL < 1 ng/mL = 2 nM. 250 mg/kg: 20% loading spray‐dried dispersion (SDD). Values represent mean ± SEM (*n* = 7).

^+^ 2.1 nM in one rat; BQL in six rats.

* Levels in two rats: 1.4 and 2.6 nM; BQL in five rats.

**TABLE 3:** Liver levels of Nezavist and DCUKA after oral administration of Nezavist.

Nezavist (nM)			
Nezavist Dose	30 min	60 min	120 min
50 mg/kg	42.1 ± 5.4	22.1 ± 2.3	18.7 ± 7.0
150 mg/kg	58.3 ± 16.3	10.2 ± 3.0	20.9 ± 5.8
DCUKA (μM)			
50 mg/kg	4.8 ± 0.5	6.7 ± 0.4	6.6 ± 0.9
150 mg/kg	5.5 ± 0.7	7.2 ± 1.2	6.6 ± 0.7

*Note:* Values represent mean ± SEM (*n* = 3).

**TABLE 4:** Brain levels of Nezavist and DCUKA after oral administration of Nezavist.

Nezavist (nM)			
Nezavist dose	30 min	60 min	120 min
50 mg/kg	BQL	BQL	BQL
150 mg/kg	BQL	BQL	BQL
DCUKA (nM)			
50 mg/kg	18 (*n* = 1)	33.1 ± 4.0	18.6 (*n* = 2)
150 mg/kg	23.3 (*n* = 2)	40.4 ± 5.8	19 (*n* = 2)

*Note:* Values represent mean ± SEM (*n* = 3 unless otherwise noted, in some instances, DCUKA values were BQL. BQL < 7.5 ng/g [15‐nM Nezavist; 16‐nM DCUKA]).

In the final set of pharmacokinetic studies, yet another formulation of Nezavist (a spray‐dried dispersion, SDD) was used. In these studies, a dose of 250 mg/kg of Nezavist was administered orally to male Wistar rats. Only two out of seven animals showed measurable levels of Nezavist in plasma at one or two time points (maximum, 1.4 and 2.6 nM), while the level of Nezavist in the other five rats was below the limit of quantification at all time points measured (Table 2).

### 3.4 Effects of Nezavist on Intestinal Contractility and Vagal Activity

When considering which organs outside of the CNS contain substantial quantities of GABA_A_ receptors, the intestine, including cells of the enteric nervous system and the enteroendocrine cells, becomes notable [29, 59]. One of the known functions of GABA in the intestine is to mediate the contraction of the intestinal musculature via GABA_A_ receptors [60], and information about the contractility of the intestine and hormonal, nutrient and immune mediator signals are conveyed to the brain from the intestine via the vagus nerve. Thus, it became of interest to examine the effect of Nezavist on intestinal function. As already mentioned, Nezavist is an effective PAM at the GABA_A_ receptor [2].

#### 3.4.1 Nezavist Effects on Intestinal Contractility

**FIGURE 9:**
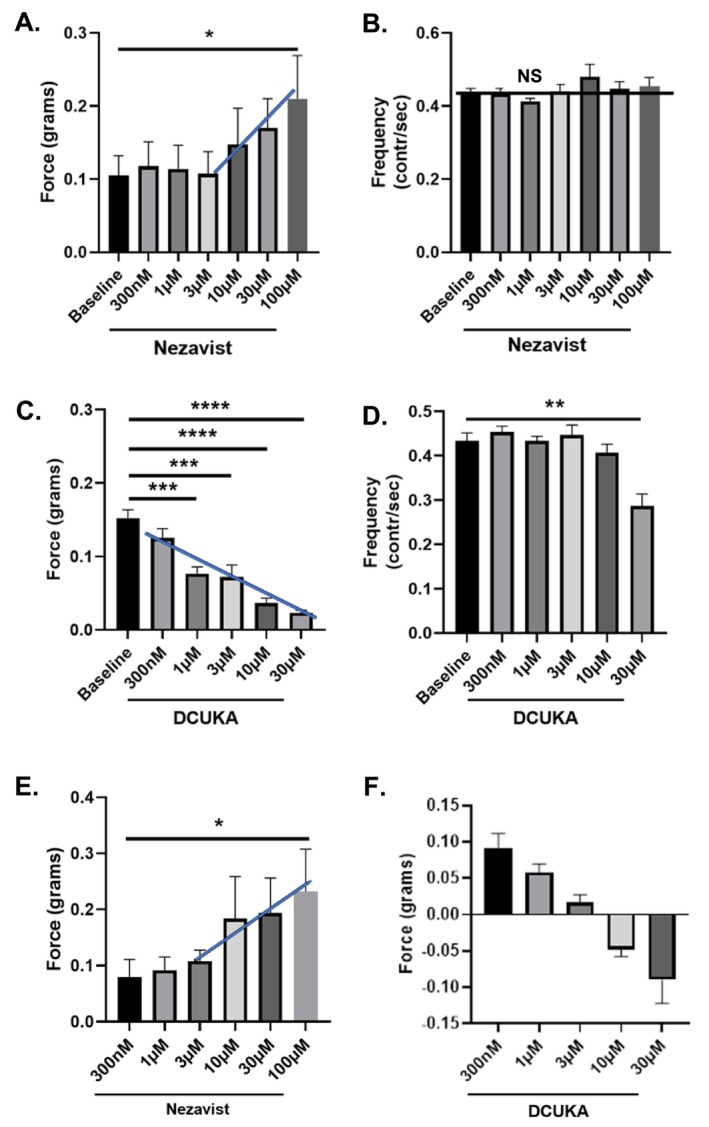
Effect of Nezavist or DCUKA on spontaneous contractility and basal tone in the ileum. Values represent mean ± SEM. (A–D) Spontaneous contraction: Force of spontaneous contraction produced by Nezavist (A) or DCUKA (C). Frequency of spontaneous contraction produced by nezavist (B) or DCUKA (D). Blue lines in subplots A and C illustrate a dose response relationship. *p < 0.05, **p < 0.01, ***p < 0.001, ****p < 0.0001 (one‐way repeat measures ANOVA followed by Tukey post hoc testing). (E,F) Basal tone: Basal tone compared to baseline ((application force—baseline tone)/(baseline tone)). Effect of Nezavist (E) or DCUKA (F) on basal tone. Blue line illustrates a dose response relationship. *p < 0.05 (one‐way repeat measures ANOVA followed by Tukey post hoc testing).

The effects of Nezavist and DCUKA on the mouse ileum and colon were examined in order to infer their potential impact on overall GI motility, as described in Seifi et al. [43, 44]. The effect of Nezavist and DCUKA on longitudinal smooth muscle contractility in the ileum was investigated by examining spontaneous contractility and basal tone. Nezavist increased the force of spontaneous contraction in a dose‐dependent manner from 3− 100 μM (Figure 9A), with a statistically significant increase from baseline at 100 μM (ANOVA and Tukey test, *p* < 0.05). The frequency of the contractions, however, was not significantly affected by the application of Nezavist (300 nM–100 μM) (Figure 9B). Interestingly, DCUKA (1–30 μM) significantly *decreased* the force of spontaneous contraction (*p* < 0.0001) and significantly decreased the frequency of spontaneous contraction at 30 μM (*p* < 0.01) (Figure 9C,D). The effect of Nezavist and DCUKA on ileal tone was also investigated (Figure 9E,F). The basal tone of the ileal tissue increased in a dose‐dependent manner following the application of 3–100‐μM Nezavist, with a statistically significant increase from baseline at 100 μM (*p* < 0.05). DCUKA had no significant effect on basal tone. The difference in effects of Nezavist and DCUKA on ileal spontaneous contraction and basal tone may reflect differences in receptor selectivity between the two compounds. For instance, Nezavist is 10 times more potent as a PAM at the GABA_A_ receptor compared to DCUKA, and DCUKA has a spectrum of activity that includes effects on other receptors [61].

In contrast to the ileum, Nezavist and DCUKA had no significant effects on spontaneous contractility or tone of the colon (data not shown, but see Section 4).

#### 3.4.2 Effect of Nezavist on Spontaneous Vagal Activity in the Intestine

**FIGURE 10:**
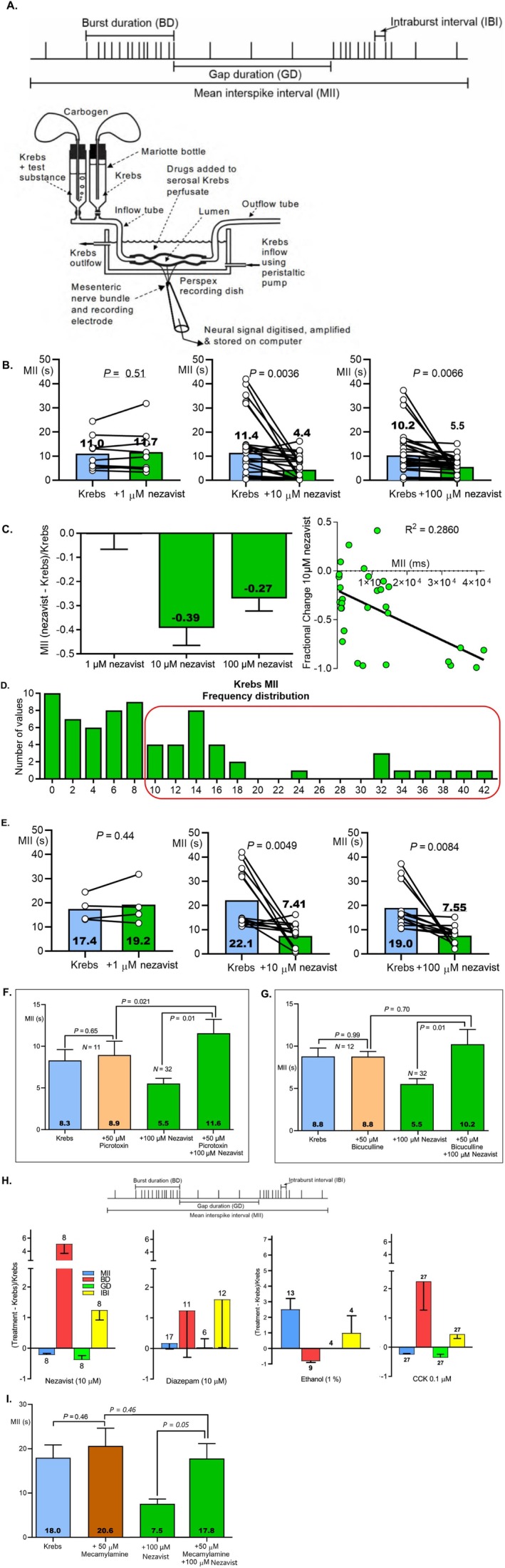
Effects of Nezavist on vagal afferent firing. (A) Gut‐to‐brain vagal afferent recording setup with mouse jejunum tissue segments. Vagal afferent signals were recorded where the mesenteric nerve bundle emerges from the small intestine. Afferent multiunit extracellular action potentials were recorded via a suction electrode from a mesenteric nerve bundle attached to a segment of the jejunum. Parameters measured from stylized single unit (action potential) firing patterns (burst duration, gap duration, intraburst interval and mean interspike interval) are illustrated in the upper diagram. (B,C) Nezavist effect on vagus mean interspike intervals (MIIs) for all MIIs obtained in the presence of vehicle (Krebs). Jejunum tissue segments were collected from male C57BL/6 mice with attached mesenteric arcade (containing a neuromuscular bundle) from which vagal nerve firing was recorded. (B) The effects of 1‐, 10‐ or 100‐μM Nezavist on MII were compared to MII in the presence of vehicle (Krebs buffer). Nezavist (10 and 100 μM) reduced MIIs (*p*‐values (displayed on each plot) were calculated by paired *t*‐test). (C) MII fractional change calculated as ((treatment—Krebs control)/Krebs control). Fractional change for 10‐ and 100‐μM Nezavist plotted against MII with a Pearson correlation coefficient of 0.3 for a best fit straight line. (D,E) Nezavist effect on vagus MIIs for vehicle MIIs > 10 s. D. Frequency distribution of MII in the presence of vehicle. In this figure, the effect of Nezavist on MIIs > 10 s, circled in red, is shown. (E) These represent slow‐firing vagal fibres, which are particularly affected by Nezavist (10 and 100 μM). *p*‐values are calculated by paired *t*‐test. (F,G) Effect of GABA_A_ receptor antagonists on the vagal firing response to Nezavist. The effect of Nezavist on MII of vagal afferent firing is illustrated. Values represent mean ± SEM *p*‐values are displayed on the plots. Neither picrotoxin (F) nor bicuculline (G) alone affected vagal firing rates, compared to vehicle (Krebs buffer). Nezavist (100 μM) significantly reduced MII (increased vagal firing), compared to vehicle (Krebs) (paired *t*‐tests with Šidák's corrections for multiple comparisons). This effect was blocked in the presence of picrotoxin or bicuculline. (H) Distinguishing vagal firing pattern codes evoked by different luminal agents. The patterns of vagal nerve firing in segments of mouse jejunum tissue were compared in the presence of Nezavist, diazepam, ethanol or cholecystokinin (CCK). Effects of these agents on mean interspike interval (MII), burst duration (BD), gap duration (GD) and intraburst intervals (IBI), as noted in the top panel, were recorded. Values represent mean ± SEM for the number of vagal fibres recorded (number above bars = *n*). Results are shown as fractional changes compared to vehicle (Krebs buffer). The GABA_A_ receptor modulator diazepam had no significant effect on any parameter, in contrast to Nezavist. The firing pattern in response to ethanol differs from that in response to Nezavist. Similar firing patterns were observed in response to Nezavist and CCK. (I) The effect of Nezavist on vagal firing rate in the presence of a nicotinic cholinergic antagonist. Mecamylamine is a broad spectrum, noncompetitive, voltage‐dependent antagonist of nicotinic acetylcholine receptors. The effect of Nezavist on MII of vagal afferent firing is illustrated. Mecamylamine alone did not significantly affect vagal firing rates, compared to vehicle (Krebs buffer). Nezavist (100 μM) significantly reduced MII (increased vagal firing), compared to vehicle (*N* = 12, Holm–Šidák's multiple comparison tests; *p*‐values on plot). This effect was blocked in the presence of a concentration of mecamylamine that produces complete inhibition of nicotinic cholinergic signalling. These data (mean ± SEM, *n* = numbers within bars) suggest a potential role for a functional vagal nicotinic sensory synapse that involves the activation of enteric nervous system intrinsic primary afferent neurons (IPANs) in the action of Nezavist.

The effects of Nezavist on vagal nerve activity were examined by the methods described in detail in West et al. [45]. Segments of the jejunum were collected from adult male C57BL/6 mice with attached mesenteric arcade containing a neuromuscular bundle and placed in Krebs buffer. An ex vivo mouse intestinal segment perfusion preparation was used to record afferent single unit vagal activity [46, 47, 48] after luminal exposure to Nezavist (Figure 10A). Nicardipine was present in the perfusion buffer during measures of electrophysiological responses. Recorded single unit events were subdivided into vehicle (Krebs) and treatment periods, and for each event, mean interspike intervals (MII) were recorded. Figure 10B illustrates the firing intensity for single vagal fibres recorded under control condition (Krebs buffer‐perfused jejunum) versus recording when 1‐, 10‐ or 100‐μM Nezavist was perfused through the jejunum. For Nezavist concentrations greater than 1 μM, MII was reduced, that is, vagal firing rates increased (paired *t*‐test, *p* = 0.0036 [10 μM], *p* = 0.0066 [100 μM]). The Pearson correlation coefficient of 0.3 for a plot of fractional change in MII versus MII values indicates a moderate strength of a linear relationship between the two variables (Figure 10C). What is notable in these data are that Nezavist dominantly stimulated the rate of discharge of a *subset* of neurons (Figure 10D,E) which, under the control (‘Krebs buffer’) condition, have a particularly slow rate of firing (high MII). This is particularly evident when a sufficient number of neurons are assessed, as can be seen in the experiments using 10 μM (paired *t*‐test, *p* = 0.0049) or 100 μM (paired *t*‐test, *p* = 0.0084) Nezavist. All of the recorded neurons are assumed to be afferent (travelling from gut to brain). Efferent neuron axons (fibres) would be quiescent, since they have been severed from the components that can generate an action potential in response to a neurotransmitter stimulus (which reside in the CNS).

Nezavist is a PAM at GABA_A_ receptors and, if GABA_A_ receptors are involved in the response noted in the firing patterns of the afferent vagal neurons, then a known GABA_A_ receptor channel blocker, such as picrotoxin [62], or a GABA_A_ receptor binding site antagonist, for example, bicuculline, would be expected to dampen or eliminate the response to Nezavist. Figure 10F,G illustrates that both picrotoxin (paired‐test, *p* = 0.01) and bicuculline (*p* = 0.01) completely blocked the effect of Nezavist, supporting the inference that Nezavist actions on vagal firing involved the GABA_A_ receptor system.

To determine if Nezavist action is unique, or whether any GABA_A_ receptor PAM would produce the same effect on vagal firing pattern codes, we compared the effect of diazepam, the classic high affinity ligand for the benzodiazepine binding site on the GABA_A_ receptor, which acts as a GABA_A_ receptor PAM [63]. Figure 10H shows that application of diazepam (at what can be considered a saturating concentration for GABA_A_ receptors) produced no significant effect on vagal afferent neuron firing patterns. One plausible explanation for this difference from Nezavist is that the GABA_A_ receptors in the gut that mediate the effect of Nezavist may contain either an *α*6 or a *δ* subunit instead of a *γ* subunit and other α subunits. Diazepam cannot function in the presence of an *α*6 or a *δ* subunit, while Nezavist can produce its PAM effect in the presence of either α6 or the *δ* or *γ* subunits [2, 63] and unpublished observation with α6‐containing GABA_A_ receptors. Ethanol (1%; a concentration expected in the upper intestine of humans after consuming alcohol [64, 65]) produced a different pattern of vagal afferent fibre firing than Nezavist (Figure 10H).

There is a report of GABA_A_ receptors containing a *δ* subunit being present on the enteroendocrine cells, which secrete cholecystokinin (CCK) in the intestine [29], and activation of GABA_A_ receptors on these CCK‐releasing enteroendocrine cells leads to membrane depolarization and potentiation of CCK release [29]. Vagal neurons contain receptors for CCK [66, 67] and thus the effects of Nezavist on vagal neuron firing may be secondary to Nezavist‐mediated release of CCK and CCK action on the vagus within the jejunal preparation. Figure 10H illustrates that exogenous application of CCK to the jejunum produced an identical vagal neuronal firing pattern code as seen with Nezavist. The similarity in the actions of CCK and Nezavist provides a plausible path by which Nezavist can activate the firing of a subset of vagal afferent neurons. Furthermore, Figure 10I shows that the effect of Nezavist on vagal firing rate can be reduced by the nicotinic cholinergic antagonist, mecamylamine (Holm–Šidák multiple comparison test, *p* = 0.05). This result suggests that Nezavist may also activate a functional vagal nicotinic cholinergic ‘sensory synapse’ that involves activity of intrinsic afferent primary afferent neurons (IPANs) in the enteric nervous system [68].

### 3.5 Effect of Nezavist on c‐Fos Levels in Mouse NTS

**FIGURE 11:**
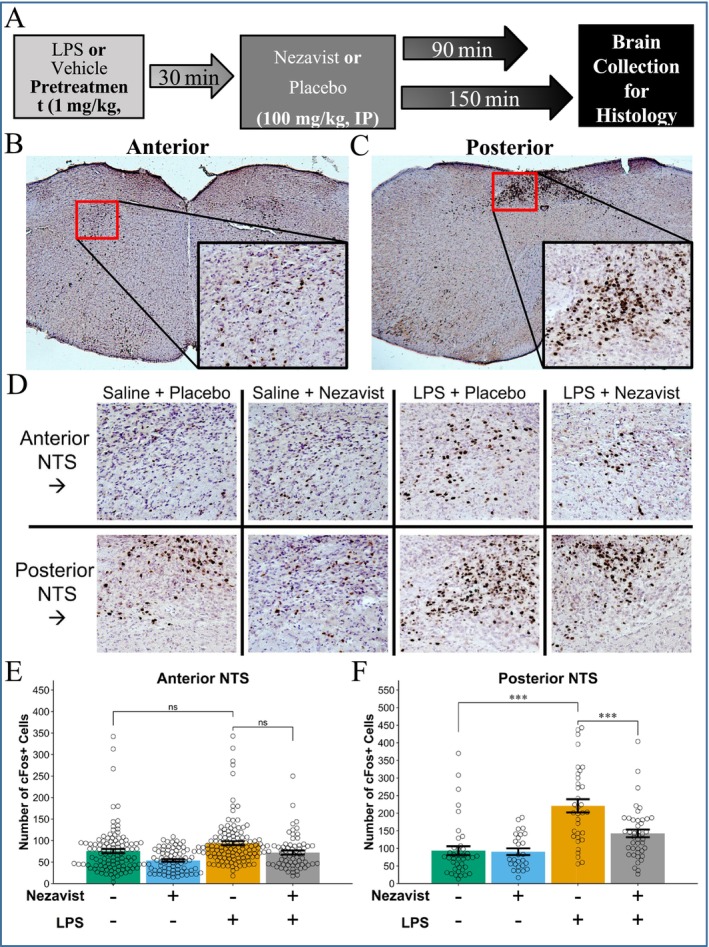
Effects of ip LPS and Nezavist on c‐Fos expression in NTS of C57BL/6 mice. (A) Experimental design of the study. (B,C) c‐Fos staining. Collected brain tissue was stained for c‐Fos and c‐Fos‐positive cells were counted as a proxy for neuronal activation. 20X magnification images were collected and analysed from both anterior (B) and posterior (C) aspects of the nucleus tractus solitarius (NTS). (D–F) Quantification of c‐Fos staining. representative images from each of the groups at the 90‐min time point are shown from anterior (D, top row) and posterior (D, bottom row) NTS. Linear mixed model analyses showed that LPS had no effect on c‐Fos‐positive cell counts in the anterior NTS (E) but significantly increased c‐Fos‐positive cells in the posterior NTS (anterior: *p* = 0.4284; posterior: *p* < 0.0001) (F). Nezavist with LPS treatment had no effect on the number c‐Fos‐positive cells in anterior NTS (*p* = 0.1235) but Nezavist significantly, but not fully, reduced LPS‐induced activation of the posterior NTS (*p* < 0.0001). There was no effect of Nezavist alone on NTS activation in either subregion (*p*s = 0.0612, anterior; 0.4555, posterior). Individual data points displayed here represent c‐Fos‐positive cell counts in individual images taken across mice. The bars and error bars represent the means ± SEMs of these images; ***, *p* < 0.001.

To investigate whether Nezavist's effect on vagal firing results in a change in vagal input into the brain, the effect of Nezavist on the expression of an immediate‐early gene, c‐Fos, in the NTS, the initial CNS target of vagal afferents, was examined. Lipopolysaccharide (LPS) is a major component of the outer membrane of Gram‐negative bacteria, which initiates a strong immune response, including an increase in inflammatory cytokines in the brain and periphery [69]. The administration of LPS and the concomitant increase in peripheral cytokines activates the vagus nerve [70]. Our experiments were designed to assess not only the effect of Nezavist on vagal signalling to brain, but also to investigate Nezavist's effect on a well‐studied c‐Fos response in the NTS produced by LPS via the vagus [71]. The experimental design is illustrated in Figure 11A. The number of c‐Fos‐positive cells was counted in mice that received pretreatment with LPS (or vehicle) and then were treated with Nezavist (or placebo). Due to the metabolism of Nezavist and timing of its behavioural effects, tissue was sampled at 90‐ and 150‐min posttreatment. Representative c‐Fos levels across an entire NTS section, and specifically within the anterior and posterior regions of interest, are shown in Figure 11B,C. The data were analysed using a linear mixed model to overcome sampling differences between mice. There was no statistically significant effect of time point (90 vs. 150 min) alone (*t*_(57.69)_ = 0.033, *p* = 0.9735) or in interaction with other factors on c‐Fos‐positive cell counts (*ps* = 0.1080–0.8653), so results are shown collapsed across time points for the anterior (rostral) and posterior (caudal) NTS. There was a significant three‐way interaction between LPS treatment, Nezavist treatment and position within the NTS on the number of c‐Fos‐positive cells (*t*_*(508.42)*_ = −4.679, *p* < 0.0001). As shown in Figure 11D–F, Nezavist alone did not alter the number of c‐Fos‐positive cells in either NTS subregion, although there was a small trend for suppression of c‐Fos‐positive cells in the anterior NTS (post hoc tests; anterior vehicle‐treated, Nezavist—placebo: *p* = 0.0612; posterior, vehicle‐treated, Nezavist—placebo: *p* = 0.45). LPS treatment, by itself, significantly increased the number of c‐Fos‐positive cells only in the posterior NTS (post hoc tests; anterior placebo‐treated LPS—vehicle: *t*_*(52.51)*_ = 0.798, *p* = 0.4284; posterior placebo‐treated LPS—vehicle: *t*_*(141.95)*_ = 8.106, *p* < 0.0001). Nezavist did not affect the number c‐Fos‐positive cells in anterior NTS when administered with LPS (post hoc tests; *t*_*(59.32)*_ = 1.562, *p* = 0.1235) but suppressed the LPS‐induced increase in c‐Fos‐positive cells in the posterior NTS (*t*_*(111.98)*_ = −4.785, *p* < 0.0001). Together, these results indicate that Nezavist significantly diminished NTS neuron activation (c‐Fos labelling increases) by LPS, specifically in the posterior (caudal) NTS.

### 3.6 Effect of Nezavist on Peripheral and Hippocampal Cytokine Levels

These experiments were designed to assess the possible downstream effects of LPS administration and the impact of Nezavist on these effects. We measured cytokine responses in blood and brain (hippocampus), and microglial activation in the hippocampus produced by LPS in the absence and presence of Nezavist treatment. In these experiments, LPS was administered ip once daily for 4 days, followed by one of two doses (50 or 100 mg/kg) of Nezavist (administered ip). None of the animals died prematurely or had to be euthanized during the course of the study.

#### 3.6.1 Behavioural Signs

Behavioural signs scores of the vehicle‐only treated control group remained low throughout the monitoring period. In contrast, all mice of the LPS treatment groups showed significantly (ANOVA, *p* < 0.001) increased behavioural symptoms (e.g., reduced general health condition and activity) following LPS treatment, reaching peak scores on Day 2. From Day 2 to Day 4, the behavioural symptoms of the LPS treatment groups steadily declined. However, both Nezavist treatment groups showed a slower rate of decline of symptoms compared to control animals (LPS, vehicle), irrespective of the administered dose. The Nezavist/LPS‐treated animals exhibited significantly higher behavioural scores on Day 4 (ANOVA and multiple comparisons, 50‐mg/kg Nezavist, *p* < 0.005; 100‐mg/kg Nezavist, *p* < 0.002) compared to the LPS/vehicle‐treated mice.

#### 3.6.2 Body Weight

All animals from the LPS treatment groups showed significant (*p* ≤ 0.008) body weight loss from Day 2 until tissue collection on Day 5, whereas the vehicle‐only treated animals maintained stable body weights throughout the entire observation period. From Days 3 to 5, the body weights of the control LPS group (LPS/vehicle) stabilized and remained constant until tissue collection, whereas the LPS/Nezavist treatment groups showed further decreasing body weights until study end. The mice that were treated with LPS and 100‐mg/kg Nezavist displayed significantly (ANOVA *p* < 0.01) lower body weights on Day 5 in comparison to the LPS/vehicle‐treated mice.

#### 3.6.3 Open‐Field Test

Compared to vehicle‐only treated controls, all LPS treatment groups showed reduced exploratory behaviour. This effect was indicated by significantly (ANOVA) reduced ‘hyperactivity’ (*p* < 0.02), total distance traversed (p < 0.02) and number and duration of rearings (*p* < 0.04). Compared to LPS/vehicle‐treated animals, the mice receiving 100 mg/kg of Nezavist and LPS showed significantly decreased ‘hyperactivity’ (*p* < 0.02) but no effects on the other measures of exploratory behaviour. No statistically significant group differences in overall activity and thigmotaxis behaviour were detected.

Defecation was assessed as a measure of emotionality and was significantly (ANOVA and multiple comparisons, *p* < 0.001) reduced in both Nezavist/LPS treatment groups in comparison to the LPS/vehicle group.

#### 3.6.4 Cytokine Levels: Hippocampus

**FIGURE 12:**
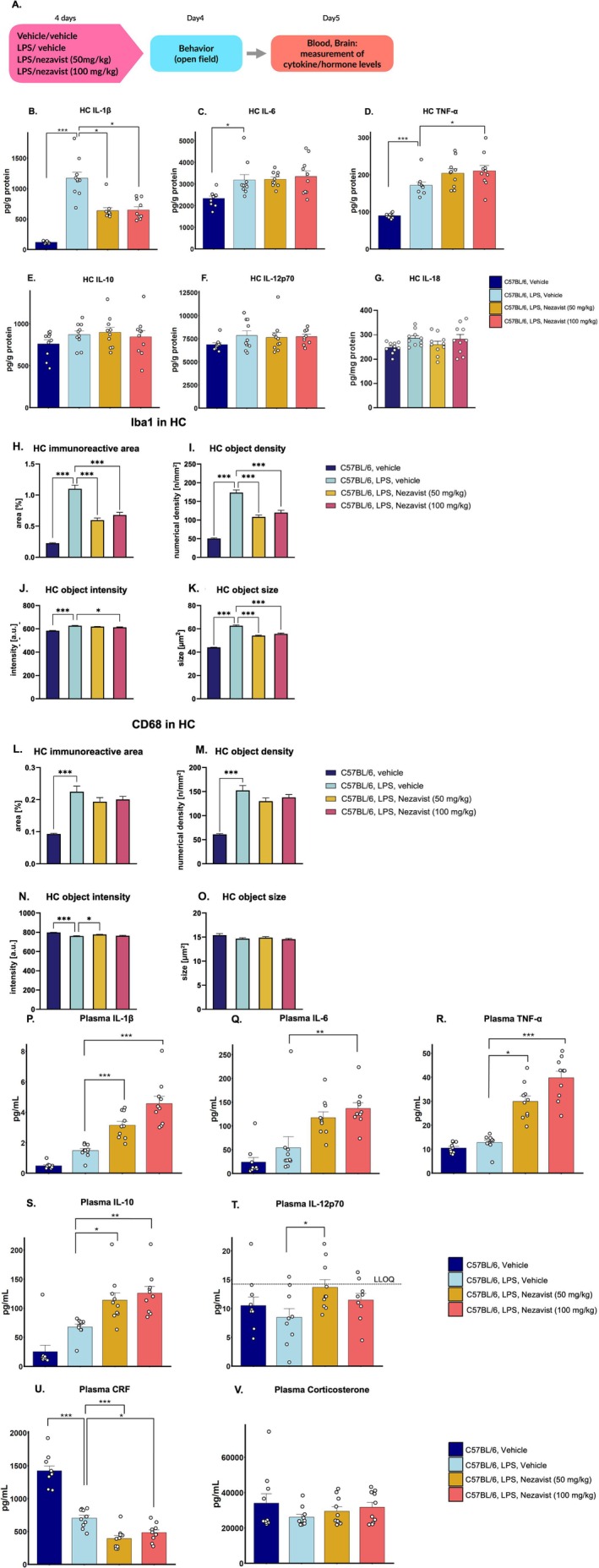
Effect of LPS and Nezavist on hippocampal cytokine levels and plasma CRF and corticosterone in mice. (A) Experimental design of the study. (B–G) Hippocampal cytokine levels. Levels of IL‐1*β* (B), IL‐6 (C), TNF‐*α* (D), IL‐10 (E), IL‐12p70 (F) and IL‐18 (G) in hippocampus collected from all treatment groups on Day 5, as determined by MSD assay, are shown (mean ± SEM). Averaged data from two separate experiments with the same samples are shown. Values are given as pg/g protein. No outlier test was performed. Statistics (*n* = 10 per group): One‐way ANOVA followed by Bonferroni's multiple comparisons test (TNF‐*α* and IL‐10) or Kruskal–Wallis test followed by Dunn's multiple comparisons test (all other measures). The C57BL/6, LPS, vehicle group served as reference group for pairwise comparisons. *p < 0.05, ***p < 0.001. (H–K) Quantification of Iba1 Immunofluorescence in the C57BL/6 Mouse Hippocampus (HC) after ip Nezavist and LPS. Immunofluorescence of Iba1 was detected with guinea pig monoclonal [Gp311H9] antibody. Iba1 immunosignal was significantly increased in LPS‐only treated animals in all four measured read‐outs when compared to vehicle‐only treated controls. Treatment with Nezavist at both doses led to significantly reduced immunoreactive area (H), density (I) and size (K) values of Iba1‐positive objects. In the case of the high dose animals (100‐mg/kg Nezavist) object intensity values were also significantly reduced (J). Graphs show the means of immunofluorescent signal on five brain sections per mouse [*n* = 10]. Data were analysed by one‐way ANOVA and Bonferroni's post hoc test. The C57BL/6, LPS, vehicle group was defined as reference group for pairwise comparisons. Bar graphs represent group means + SEM. *p < 0.05, ***p < 0.001. (L–O) Quantification of CD68 Immunofluorescence in the C57BL/6 mouse hippocampus (HC) after ip Nezavist and LPS. Immunofluorescence of CD68 was detected with rat monoclonal [FA‐11] antibody. The immunosignal was significantly increased in the LPS‐only treated animals when compared to vehicle‐only treated controls. Slightly lower mean values of immunoreactive area (L) and object density (M) were found in mice that were treated with Nezavist; however, treatment effects were only significant in the case of the object intensity (N) in the lower dose (50 mg/kg) Nezavist‐treated animals. Graphs show the means of immunofluorescent signal on five brain sections per mouse [*n* = 10]. Data were analysed by one‐way ANOVA and Bonferroni's post hoc test (object intensity and object size) or by Kruskal–Wallis test and Dunn's post hoc test (immunoreactive area and object density). The C57BL/6, LPS, vehicle group was defined as reference group for pairwise comparisons. Bar graphs represent group means ± SEM. *p < 0.05, ***p < 0.001. (P–T) Effects of LPS and Nezavist on plasma cytokine levels in C57BL/6 mice. Levels of IL‐1*β* (P), IL‐6 (Q), TNF‐*α* (R), IL‐10 (S) and IL‐12p70 (T) in terminal plasma samples collected from all treatment groups on Day 5, as determined by MSD assay, (mean ± SEM). Data are given as pg/mL plasma. No outlier test was performed. Statistics (*n* = 9–10 per group): One‐way ANOVA followed by Bonferroni's multiple comparisons test (IL‐1*β* or IL‐12p70) or Kruskal–Wallis test followed by Dunn's multiple comparisons test (all other measures). The C57BL/6, LPS, vehicle group served as reference group for pairwise comparisons. *p < 0.05, **p < 0.01, ***p < 0.001. LLOQ, lower limit of quantification. (U,V) Effects of ip LPS and Nezavist on plasma CRF and corticosterone in C57BL/6 mice. Levels of CRF (U) and corticosterone (V) in terminal plasma samples collected from all treatment groups on Day 5, as determined by ELISA, are shown (mean ± SEM, pg/mL plasma). No outlier test was performed. Statistics (*n* = 10 per group): One‐way ANOVA followed by Bonferroni's multiple comparisons test (CRF) or Kruskal–Wallis test followed by Dunn's multiple comparisons test (corticosterone). The C57BL/6, LPS, vehicle group served as reference group for pairwise comparisons. *p < 0.05 and ***p < 0.001.

Cytokine levels in the hippocampus were measured at 18 h after the last LPS or LPS/Nezavist treatment. Compared to vehicle‐only treated control mice, the LPS/saline‐treated mice showed significantly (ANOVA) increased levels of hippocampal IL‐1*β* (*p* < 0.001), IL‐6 (*p* < 0.05) and TNF‐α (*p* < 0.001) (Figure 12B–D).

In the hippocampus, the treatment with Nezavist at both of the tested doses given after LPS had no significant effect on IL‐6 increases produced by LPS but significantly reduced the LPS‐generated hippocampal IL‐1*β* levels (by approximately 45%, *p* < 0.05), compared to LPS/vehicle‐treated mice. Nezavist (100 mg/kg) produced a very small but significant (*p* < 0.05) increase in TNF‐*α* levels, compared to LPS/vehicle‐treated mice. Neither LPS nor Nezavist plus LPS altered the levels of IL‐10, IL‐12p70 or IL‐18 in hippocampus (Figure 12E–G).

#### 3.6.5 Hippocampal Histology

Brains were obtained at 18 h after the last LPS or LPS/Nezavist treatment for histological analysis. Brains were stained for Iba1 (Ionized calcium‐binding adaptor molecule) (1), a marker that is particularly useful for identifying activated microglia and macrophages [72], GFAP (glial fibrillary acidic protein), a marker for astrocytes [73], and CD68, which identifies infiltrating monocytes and macrophages, and is also used to identify activated microglia [74]. For quantification, the region of interest (hippocampus) was identified, and the three proteins were quantified based on (1) immunoreactive area, to determine overall differences in immunoreactivity across treatment groups; (2) the number of objects, normalized to the region of interest (object density); (3) mean signal intensity of identified objects, which indicates whether there are differences in cellular expression of the various proteins across treatment groups; and (4) object size. Figure S2 shows the immunofluorescence of the measured proteins. The effects of LPS and Nezavist on Iba1 and CD68 staining in hippocampus are illustrated in Figure 12H–K and L–O, respectively. LPS increased the hippocampal immunoreactivity, object density, object intensity and object size of Iba1 (ANOVA *p* < 0.001). Nezavist at both doses reduced the LPS‐induced increases in immunoreactivity, density and size of objects (*p* < 0.001), and at the higher dose also significantly reduced object intensity (*p* < 0.05, Figure 12H–K). LPS treatment increased hippocampal immunoreactivity and object density of CD68 (*p* < 0.001) and produced a very small but statistically significant reduction in object intensity (*p* < 0.05), with no significant effect on object size (Figure 12L–O). Although treatment with both doses of Nezavist reduced the effect of LPS on immunoreactive area and object density of CD68, these changes did not reach statistical significance. 50‐mg/kg Nezavist produced a small but statistically significant increase in CD68 object intensity (*p* < 0.05) (Figure 12N). LPS had little effect on staining for GFAP, and Nezavist did not affect the response to LPS (data not shown). Overall, these results indicate a substantial and significant effect of Nezavist to reduce the LPS‐induced increase in Iba1 microglial staining. While CD68 staining is less selective for identifying activated microglia and also identifies monocytes and macrophages infiltrating from the periphery, the trend is similar for the effect of Nezavist to reduce the hippocampal CD68 response to LPS.

#### 3.6.6 Cytokine Levels in Plasma

In contrast to the effects in hippocampus, treatment with Nezavist and LPS led to a significant (ANOVA) and dose‐dependent *increase* in the plasma levels of IL‐1*β*, IL‐6, TNF‐*α* and IL‐10 (*p* < 0.05 to 0.001), compared to mice that received the LPS/saline treatment. Furthermore, a significant (*p* < 0.05) increase in the plasma IL‐12p70 levels was detected in mice that were treated with 50‐mg/kg Nezavist and LPS, while LPS/saline had no effect (Figure 12P–T).

#### 3.6.7 Plasma CRF and Corticosterone Levels

Compared to vehicle‐only treated animals, plasma CRF levels of the LPS/saline‐treated mice were significantly reduced by approximately 50% when measured 18 h after the last dose of LPS (*p* < 0.001). Treatment with Nezavist plus LPS at either of the tested doses of Nezavist led to an even further reduction of the plasma CRF levels (50 mg/kg, *p* < 0.001; 100 mg/kg, *p* < 0.05, Figure 12U). In contrast, there were no statistically significant changes in plasma corticosterone levels produced by LPS/saline or LPS plus Nezavist treatment (Figure 12V).

## 4 Discussion

AUD is a medical condition characterized by an ‘impaired ability to stop or control alcohol use despite adverse social, occupational or health consequences’ (http://NIAAA.NIH.gov/publications/brochures‐and‐fact‐sheets/understanding‐alcohol‐use‐disorder). The inability to stop alcohol use has led to the definition of AUD as a chronically relapsing condition wherein attempts at sobriety are curtailed by return to chronic consumption of large amounts of alcohol (‘relapse behaviour’). Given this characterization and definition of AUD, we have focused our medication development efforts on therapeutics that would prevent or diminish relapse in individuals who attempt to reduce their ‘dependence’ on alcohol by embarking on periods of abstinence or diminished consumption.

### 4.1 Nezavist Effect in Animal Models of Abstinence‐Induced Escalation of Alcohol Consumption

There is a rich literature on animal models for studying alcohol relapse [75, 76]. The described models provide both face validity and predictive validity for discovery of medications that can decrease drug relapse in humans. We have exploited two highly utilized animal (rat) models of relapse behaviour in AUD to assess our candidate drug, Nezavist. The first model is one described by Spanagel et al. [58] in which animals consuming alcohol for several months are deprived of their source of alcohol and after a period of days or weeks are again allowed to consume alcohol. In this model, with the reintroduction of alcohol, the animals exhibit ‘relapse‐like’ drinking (significantly increased consumption of alcohol), and the animals demonstrate an ‘incentive demand’ to consume alcohol [32, 33].

The second model of alcohol dependence and relapse in our studies utilized a procedure in which (1) animals are trained to respond for alcohol in an operant paradigm; (2) animals are then exposed intermittently to alcohol vapour to the point of showing dependence, i.e., alcohol withdrawal signs when alcohol exposure is terminated; and (3) after 10 h of withdrawal, animals are returned to the operant chamber to assess their responding for alcohol solution versus water [38, 39].

In the current studies, both models performed as expected. There was an escalated consumption of alcohol solution when the dependent rats were offered a choice between drinking alcohol or water after a prolonged period (months) of voluntarily consuming alcohol in a free‐choice paradigm followed by alcohol deprivation [32] (see Figure 1). Similarly, there was significantly greater responding on the alcohol delivery‐associated lever in the operant responding paradigm after withdrawal from dependence‐inducing chronic alcohol exposure [37, 38, 39] (see Figure 2). Each of these models has previously been extensively used to test medications already known to reduce craving in abstinent humans recovering from AUD (e.g., Acamprosate or naltrexone/nalmefene), providing evidence for predictive validity [57, 77, 78, 79]. When we administered Nezavist (20 mg/kg × 5, ip, over a 3‐day period) to ethanol‐deprived rats in the model described by Spanagel and colleagues [32, 33], we noted diminished total alcohol consumption and an increase in water consumption, which resulted in a significant decrease in ‘alcohol preference’ during the period of Nezavist administration.

The further conclusion that can be generated from results of the analysis of Nezavist effects on consumption of particular concentrations of alcohol is that Nezavist in low doses (20 mg/kg/dose) changed the preference of the alcohol‐dependent animals from a higher to a lower concentration of alcohol. Wistar‐derived rats, as those used in these studies, have been reported to have a preference for 10% solutions of alcohol (ethanol) when offered solutions of various concentrations [80]. The alcohol deprivation effect in the alcohol‐dependent rats demonstrates that, without drug treatment, the rats will consume the most alcohol by drinking the solution with the highest alcohol concentration (20%). The administration of the low dose of Nezavist appears to selectively lower the amount of alcohol ingested by drinking the high concentration of alcohol, but there appears to be a partial compensation by an increased consumption of the preferred 10% alcohol solution. The result is a modest reduction in *overall* (total) consumption of alcohol on the first day of abstinence and an overall increase in water consumption, but not total fluid consumption.

One can consider the dose of 20‐mg/kg Nezavist, given ip five times over a 3‐day period, as a minimum effective dose in this multi‐dose paradigm, and 75 mg/kg (also given ip five times over 3 days) as a maximum (possibly supramaximal) dose for reducing relapse‐like alcohol consumption. Although the animals receiving the 75‐mg/kg dose significantly decreased consumption of all concentrations of alcohol and increased water consumption, there was a decrease in locomotor activity measured during the first 3 days of the high dose Nezavist administration. On the other hand, the locomotor activity was similar on Days 4–7 between the Nezavist and vehicle‐treated rats, but the Nezavist‐treated rats consumed minimal or no alcohol and still maintained high levels of water consumption. No evidence of Nezavist accumulation in the circulation of rats treated with multiple doses of Nezavist was evident in our pharmacokinetic assessments. One conclusion that can be drawn from the effects of Nezavist on the abstinence‐induced alcohol consumption is that a low dose of Nezavist produces an aversion for higher concentrations of alcohol, while the high dose generates an aversion to alcohol across all the tested concentrations, and this aversion is long‐lasting and maintained beyond the presence of Nezavist or its major metabolite within the body.

Acamprosate (calcium‐bis(*N*‐acetylhomotaurinate)) is one of the few pharmacotherapies approved for treating AUD (reducing propensity for relapse) in humans [81]. Although there is controversy regarding which component of acamprosate, the calcium or homotaurine, or the combination of both, is required for reducing relapse [82, 83], the calcium‐containing product, i.e., calcium‐bis(*N*‐acetylhomotaurinate), was used in our studies. Acamprosate was also previously tested in both of the models used in our work and produced a diminution in the relapse‐like alcohol consumption and operant responding for alcohol [57, 77]. We considered that acamprosate would be an appropriate comparator for Nezavist. The prior studies indicated that multiple doses of Acamprosate were more effective than a single dose [77] and thus we used multiple doses (5 × 200 mg/kg doses over a 3‐day period) of Acamprosate for comparison with multiple (5× 20 mg/kg) doses of Nezavist. The effects of Nezavist and Acamprosate were similar, with Nezavist (20 mg/kg/dose) reducing alcohol consumption by 16% during the first day of alcohol consumption after the deprivation period, and acamprosate (200 mg/kg/dose) producing a 21% reduction. Although more work is required, Nezavist may be superior, on a dose basis, to acamprosate.

The operant responding model, a negative reinforcement model of relapse [6, 7] allowed for a further characterization of Nezavist actions. In this paradigm, we established that a single dose of Nezavist, given ip, a short time (60 min) prior to testing for operant responding for alcohol by an alcohol‐dependent animal, after a period of alcohol withdrawal, could reduce alcohol dependence‐induced drinking (i.e., the significant increase in responding for alcohol). Nezavist produced a dose‐dependent reduction in responses for the alcohol solution. The 50‐mg/kg dose returned responding to baseline levels recorded prior to instituting the ‘deprivation’ period. In this model, we also ascertained that the initial and primary metabolite of Nezavist, DCUKA, when given ip at the same dose as Nezavist, produced no effect on the ‘relapse’ phase of responding for alcohol or water. When Nezavist was given orally a higher dose was necessary to significantly reduce the dependence‐induced increase in alcohol responding. In this case, there was a significant increase in responding for water that accompanied the decrease in responding for alcohol. The effect of a single dose of Nezavist (administered orally) abated within 4 h after administration of Nezavist to rats. The efficacy of Nezavist on responding for alcohol by animals *not* made dependent on alcohol was significantly lower than the effect in the alcohol‐dependent rats, suggesting that a neurobiological difference is present in the dependent/withdrawn versus non‐alcohol‐dependent rats.

### 4.2 Effect of Nezavist on Other Alcohol‐Related Behaviours

Alcohol relapse in humans is driven by a number of factors. Stress is a major factor that drives relapse [84] and withdrawal or deprivation from alcohol in alcohol‐dependent humans or other animals elicit stress‐like states [85, 86]. Exaggerated responses to stress during abstinence can be exhibited as hyperkatifeia, such as mood disturbances, anxiety and irritability, that combine with learned expectations of subjective actions of a drug like alcohol to drive compulsive alcohol seeking [7].

We tested Nezavist effects in animal models of anxiety, sedation/incoordination and stress coping strategies to ascertain the possible role of effects on these activities that could be related to the actions of Nezavist on alcohol relapse behaviour. Nezavist showed no effect in models of anxiety‐like behaviours, and there was little or no effect on locomotion or incoordination caused by single doses of Nezavist well above those producing effects on relapse. However, a significant effect (reduced immobility) was noted on female rat behaviour in the forced swim test (Porsolt test). The Porsolt test [42] has been used extensively for ascertaining an animal's coping strategy to an acute stress [87]. It may seem that a drug that generates a signal in a test of an animal's coping strategy to an acute stress [87] would also produce some signal in tests of anxiolytic properties. However, it has been well demonstrated that particular agents can be distinguished in their actions by tests for anxiolytic effects (elevated plus maze, open‐field activity, etc.) or effects in the forced swim test [88, 89]. A recent review by Molendijk and deKloet [90] provided a summary of mechanistic and anatomical data relevant to the determinant of immobility time in the forced swim test. They emphasized the relationship of the behavioural response to glucocorticoid actions on ‘circuits processing salient information … and memory consolidation’, and activity of the medial prefrontal cortex and the periaqueductal grey (PAG) with modulation by the NTS. Given that the NTS is the relay station between the cholinergic input to the brain via the vagus nerve and the noradrenergic output to other brain areas such as the PAG and hippocampus (see below), the forced swim test may reflect Nezavist actions that involve the aforementioned brain areas and the immune system influence of the glucocorticoids.

We also tested the effects of Nezavist on the metabolism of alcohol (ethanol) in mice and the effect of Nezavist on the sedation and incoordination produced by alcohol. Circulating levels of alcohol in mice after a dose of 3.5 or 1.5 g/kg of alcohol were not affected by prior treatment with Nezavist (50 or 200 mg/kg ip), and there was no difference in the sedative/hypnotic (sleep time) or incoordinating effect of alcohol between animals pretreated with Nezavist or vehicle. These results indicate that the effects of Nezavist on alcohol consumption/relapse‐like behaviour are not due to alterations in alcohol metabolism or a change in alcohol's sedative or incoordinating effects.

### 4.3 Pharmacokinetic Studies of Nezavist and its Metabolite, DCUKA

Given the time course of the effect of a single dose of Nezavist on operant responding for alcohol in the alcohol‐dependent animals, we pursued pharmacokinetic studies to ascertain the relationship of blood and tissue (including brain) levels of Nezavist and the behavioural effects. Surprisingly, we found little or no Nezavist in peripheral blood or brain at any time after oral or ip administration of Nezavist at doses commensurate with those producing behavioural effects. Of the three biological entities that were tested, the one organ with notable Nezavist levels was the liver. These results indicate that Nezavist, absorbed through the intestine, is subject to extensive first‐pass metabolism by the liver, leaving little or no Nezavist to reach other organs. This interpretation is supported by the fact that the Nezavist metabolite, DCUKA, was evident in the circulation and was increased in a dose‐dependent manner with increasing doses of Nezavist. It should be noted that the absorption of drugs, such as Nezavist, after ip injection occurs by way of mesenteric arteries in the peritoneum and follows a path through the blood supply to the small intestine to the portal vein entering the liver [91]. Drugs injected ip are subject to first‐pass metabolism by the liver after they pass through the blood vessels of the intestine. Thus, both oral administration and ip injection of Nezavist would access the blood supply of the intestine prior to reaching the liver, albeit concentrations within the intestine may be different given the two routes of administration, due to absorption characteristics and metabolism in the intestine. Nezavist is metabolized to DCUKA by carboxylesterase (it is a preferred substrate for Carboxylesterase 1; data not shown). In the rat, Carboxylesterase 1 is expressed at high levels in blood and liver (the human differs from the rat by the fact that little Carboxylesterase 1 is found in blood). However, initial pharmacokinetic studies with humans indicate that little or no Nezavist is found in the peripheral circulation, but DCUKA is present, similar to what is found in the rat (data not shown). Thus, first‐pass metabolism in the liver may play an important part in determining the circulating levels of Nezavist in humans and rats. Given that little or no Nezavist is present in the circulation, it is not surprising that Nezavist is not found in the brain of rats given doses of Nezavist that are active in behavioural studies. Measurable levels of DCUKA are found in rat brain after administration of Nezavist, but the administration of DCUKA (50 mg/kg) per se produced no effect on dependence‐induced escalation of alcohol intake. The data on the essentially null circulating and brain levels of Nezavist led us to seek a location where Nezavist could be present outside the CNS and yet generate a behavioural response.

### 4.4 Nezavist Effects on Intestinal Motility and Vagal Nerve Firing

The stomach and intestines are highly innervated by the vagus nerve [92], which carries sensory information to the CNS (primarily to the NTS). Ample evidence has been presented that vagal input to the CNS can communicate information to the brain about the intestinal microbiome, nutritional factors (glucose, fatty acids and amino acids), distention of the intestine, hormones controlling hunger and satiety, inflammatory mediators, and so forth. Electrical stimulation of the vagus (ESV) has been shown to be effective in controlling epileptic seizures and improving drug‐resistant major depressive disorder and is approved in the USA by the FDA for treating these disorders [93]. More recently, ESV has been shown to have efficacy in treating addictive disorders [94, 95, 96]. The most effective application of ESV for long‐term treatment is through implanted electrodes and this surgery, and accompanying discomfort of the implant has limited the application of this therapy [97, 98]. A pharmacological approach to vagal stimulation could be beneficial.

We decided to test Nezavist actions on the activity of afferent vagal neurons in an in vitro preparation utilizing the small intestine of the mouse. However, given the fact that Nezavist has been characterized as a PAM at the GABA_A_ receptor [2], we first tested Nezavist actions on physiological functions of the gut known to involve GABA. The enteric nervous system controls peristaltic activity of the small intestine [60]. Cholinergic activation of GABA interneurons generates the release of GABA onto GABA_A_ receptor‐expressing cholinergic motor neurons that control intestinal tone and contraction. If Nezavist acts in the intestine, one would, thus, expect some effect of Nezavist on gut tone or contractility. Using in vitro preparations of mouse ileum and colon, we assessed the effects of Nezavist on gut contractile properties [43, 44]. Since isolated intestinal segments display spontaneous contractions, we expected that there would be endogenous release of GABA, and therefore, we did not add exogenous GABA_A_ receptor agonists to perfusion fluids in our studies. It should be noted that GABA acts as an *excitatory* neurotransmitter in the gut [59], and Nezavist produced effects in the ileum preparation that would be expected from potentiation of GABA actions at the GABA_A_ receptor in this preparation. There was little effect of Nezavist in the preparation of the colon. This can be explained by the distribution of GABA_A_ receptors along the digestive tract, with a high concentration of these receptors in the upper GI tract and sparse expression in the colon [99].

The question remained as to whether Nezavist could generate afferent signalling in neurons of the vagus nerve not directly related to the GABA‐mediated effects on tone and force of contraction in segments of the upper GI tract. To examine this possibility, we used a preparation of the mouse jejunum but paralysed muscle contraction with nicardipine. Electrical activity of vagal neurons emanating from the segment of jejunum was measured by the well‐described methods of West et al. [45]. The results presented in Figure 10 clearly indicate that the addition of Nezavist to the perfusion fluid changes the firing properties of vagal neurons and particularly *enhances* the firing of a set of slowly discharging neurons. These particular neurons have long periods between action potentials under control conditions, and these neurons increased discharge rates in the presence of Nezavist. In our study, this pattern was also mimicked by cholecystokinin (CCK), but the Nezavist pattern was quite different from that produced by alcohol (ethanol) at concentrations expected in the intestine of humans after consumption of various forms of alcohol (beer, wine and distilled beverage) [64, 65] or by the Gram‐negative bacterial antigen LPS [45].

If Nezavist is acting as a PAM at GABA_A_ receptors, then aside from the GABA involvement in gut muscle contraction, one wonders about the source of GABA and location of GABA_A_ receptors instigating the changes in discharge properties of vagal afferent neurons. There is a modicum of evidence that afferent vagal neurons may express GABA_A_ receptors (in nodose ganglion cell bodies [31, 100, 101]), and these receptor subunits may be transported to sensory terminals, but indirect mechanisms may also contribute to Nezavist actions. For instance, there is good evidence that GABA_A_ receptors are located on enteroendocrine cells that synthesize and release CCK [29]. Activation of these GABA_A_ receptors promotes the release of CCK, and CCK can interact with the CCK‐1 receptor on vagal sensory endings to change vagal neuron firing properties [66, 67]. As demonstrated (Figure 10), CCK produces a similar pattern of changes as Nezavist on vagal neuron firing. The other possibility is that Nezavist is acting within the enteric nervous system to generate signals that are then transmitted to vagal neurons [68]. As already mentioned, the enteric nervous system does express GABA_A_ receptors and utilizes GABA as a transmitter. If there are GABA‐initiated signals that can be transmitted from the enteric nervous system to the vagus neurons [68], Nezavist can be potentiating this pathway. For example, a pathway involving sensory function of IPANs within the enteric nervous system has been identified using the GABA‐synthesizing *Lactobacillus rhamnosus* [68, 102]. Also, IPANs express GABA_A_ receptors whose activation by GABA evoked excitatory inward currents in IPANs due to the high intracellular Cl^−^ ion concentration present within primary sensory neurons leading to a positive Cl^−^ reversal potential [103]. Transmission of this sensory information to the vagus via a nicotinic (*α*7 subunit‐containing) cholinergic synapse has been described [68]. This pathway may well explain the observation that the effects of Nezavist on vagal neuron discharge can be blocked by the nicotinic cholinergic antagonist, mecamylamine (Figure 10).

### 4.5 Nezavist Effects on Neuronal Activity in Mouse NTS

The expectation then exists that the activation of a subset of afferent vagal neurons can be reflected by changes in the c‐Fos responses in target neurons in the NTS. During the last decade, it has become popular to invoke a link between inflammation, the intestine and the brain in the aetiology of AUD [12, 104, 105]. Chronic consumption of large quantities of alcohol produces dysbiosis in the intestine and increased permeability of the intestinal lumen [9, 106]. These events allow for the entry of bacterial products (e.g., LPS) that can activate the peripheral innate inflammatory system [9, 11], and cytokines released peripherally can alter brain function, contributing to the development of AUD. A rapid route for cytokine signalling to the brain is via the vagus nerve input into the NTS. We considered that the ip administration of the LPS could mimic the effect of chronic alcohol exposure on immune signalling to the brain, and that we could gain insights from assessing Nezavist actions on the effects of LPS.

The administration of Nezavist alone, to mice, did not produce any significant changes in c‐Fos expression in the NTS, but the administration of Nezavist with LPS significantly *reduced* the c‐Fos response to LPS, particularly in the caudal region of the NTS (Figure 11). This result suggested that the effect of Nezavist‐induced changes in vagal neuron firing rates in the isolated jejunum segments may be only visible in the brainstem nuclei under conditions when another stimulus (LPS or chronic alcohol?) is producing a significant enhancement of c‐Fos expression in the NTS. It is important to note that acute administration of alcohol to rats does produce an increase in c‐Fos expression in several brainstem regions including the NTS, and this increased c‐Fos expression is to a large extent localized to the catecholamine and NPY‐producing NTS neurons [107]. However, as we determined, the acute alcohol‐generated signals from the vagal afferents did not resemble the signal produced by Nezavist. This would lead one to believe that Nezavist is not a *substitute* for acute alcohol consumption. However, given the effect of chronic alcohol consumption on the intestinal microbiome [11, 104, 105], the vagal response to chronic alcohol exposure may be expected to differ from the acute response to alcohol

### 4.6 How Does Vagal Activation by Nezavist Reduce the Effect of LPS on NTS Neuron Activity?

Jin et al. [71] examined the targets of the vagal pathways responding to LPS in the NTS of mice and identified several clusters of cell bodies in the caudal regions of the NTS (cNTS) that respond to peripherally administered LPS. The responding neurons were identified as belonging to glutamatergic and GABAergic neuron types. The chemogenetic activation of the glutamatergic neurons produced a significant diminution in the LPS‐induced peripheral cytokine response (an NTS‐mediated cholinergic anti‐inflammatory reflex) [108, 109], while the chemogenetic activation of the GABA neurons in the cNTS produced no significant effect on the peripheral levels of cytokines measured after LPS administration. The identified glutamatergic clusters of the cNTS were also found to contain noradrenergic cell bodies expressing dopamine‐*β*‐hydroxylase (DBH), and selective activation of the DBH‐expressing neurons also resulted in suppression of the LPS‐induced increase in proinflammatory cytokines in blood and an increase in IL‐10 (anti‐inflammatory cytokine) levels. Correspondingly, the ablation of the cNTS noradrenergic neurons resulted in an *enhancement* of the LPS‐generated increase in proinflammatory cytokines in the periphery and diminution of IL‐10 levels [71]. Chen et al. [110] also clearly identified noradrenergic neurons with cell bodies in the cNTS, and in their studies, chemogenetic *activation* of DBH‐containing neurons in the cNTS showed *inhibition of feeding* behaviour in fasted mice (another facet of NTS noradrenergic neuron function). On the other hand, chemogenetic *inhibition* of the noradrenergic neurons in the cNTS resulted in a *block* of the noradrenergic neuron action on feeding behaviour. The studies of Chen et al. [110] and Jin et al. [71] demonstrate that the noradrenergic neurons of the cNTS are not singular in their function and various subsets of these neurons may relate to different measured outcomes. Projections of the cNTS noradrenergic neurons reach the hypothalamus, the central nucleus of the amygdala (CeA), the bed nucleus of the stria terminalis, the locus coeruleus and so forth [111]. Thus, the noradrenergic neurons of the NTS may not only differ in their anatomical targets but also the effect of their input to the targeted areas may well depend on whether these noradrenergic neurons are activated or inhibited within the NTS.

The work of Jin et al. [71] further discerned that the vagal response to LPS was not a result of direct stimulation of the vagal neurons by LPS but was dependent on LPS‐mediated release of cytokines from the immune cells of the small intestine. It was also found that proinflammatory and anti‐inflammatory (IL‐10) cytokines each activated a different, small, subset of non‐overlapping vagal neurons. The cytokine‐activated vagal neurons made monosynaptic connections with DBH‐expressing neurons in the cNTS. The cytokine‐specific responses of particular vagal neurons have also been examined by others [112] and their findings indicate that the vagal neurons responding to a particular cytokine can be distinguished not only by their biochemical characteristics (e.g., the type of expressed receptors, enzymes and transporters [71]) but also by their firing patterns. In this regard, our results do indicate that the Nezavist‐sensitive vagal fibres display a distinguishable firing pattern [45].

As noted, GABAergic neurons are also present in clusters throughout the NTS and a significant number of the GABA neurons receive monosynaptic input from vagal afferents [113, 114]. The major vagal input to the GABA neurons in the NTS is from the intestines, and GABA neurons in the NTS are primarily interneurons [114], with a small number projecting to other brain areas [115, 116]. Acting as interneurons in the NTS, the GABA neurons have been shown to synapse with noradrenergic neurons of the cNTS (A2 noradrenergic cell‐containing nucleus) [117] and inhibit cNTS neuron function after their activation by vagal input.

One of the key observations resulting from our studies with Nezavist was that Nezavist *activated* a *subset* of afferent vagal neurons in the intestine, and the c‐Fos expression elicited by LPS in the NTS was diminished—not potentiated—by Nezavist. The afferent vagal input to the NTS can synapse with a particular set of GABAergic interneurons, which in turn inhibit a larger population of *non‐GABAergic neurons* in the NTS [114]. In our analysis, it was neurons in the caudal NTS that showed the diminished c‐Fos response to LPS when Nezavist was administered. The work of Thek et al. [114] provides evidence that the GABAergic interneurons generating inhibitory effects in the NTS may also contain somatostatin and they suggest that there may be a small number of such neurons, but they affect a large number of downstream cells. The visualization of Nezavist's effect on c‐Fos may be contingent on activation of these particular GABA interneurons by Nezavist treatment. Given the work of Jin et al. [71], a logical candidate neuron in the pool of NTS neurons activated by LPS, and susceptible to the inhibitory effect of Nezavist via a GABA interneuron, is the DBH‐containing neuron. A reason for the lack of effect of chemogenetic activation of GABA neurons in the NTS on immune responses in the work of Jin et al. [71] may have been the location of the GABA neuron clusters activated in their studies. Their work concentrated on the anatomically defined caudal NTS, and the GABA neuron clusters of interest could occur more anterior to the region that they examined [114]. It is evident from the work of Jin et al. [71] and others [118, 119] that lesioning or chemogenetically inhibiting the DBH‐containing neurons in the caudal NTS exacerbates the peripheral immunological response to LPS and modifies other responses (e.g., CCK‐induced anorexia). It has already been mentioned that NE neurons emanating from the cNTS are widely distributed throughout the CNS and participate in or affect *not only* the cholinergic anti‐inflammatory circuit [71] and response to peripheral hormones controlling appetite [110, 120] but also modulate the responses to stressful stimuli and participate in control of emotional and cognitive processing [118]. Therefore, Nezavist effects on ‘relapse‐like’ alcohol consumption that we have demonstrated can originate in the gut, be transmitted to the CNS by the afferent vagal system, activate inhibitory GABA neurons in the NTS and diminish the function of noradrenergic neurons emanating from the NTS region in the brainstem. Which particular noradrenergic projection is most important for the effect of Nezavist on alcohol consumption is yet to be determined, but our studies of the Nezavist effect on immune signalling within the brain and in the periphery do provide some evidence related to the sites of action of Nezavist.

### 4.7 How Does Vagal Activation by Nezavist Affect the Peripheral and Central Cytokine Response?

Our results with measures of peripheral cytokine levels after LPS administration, and the effects of LPS in conjunction with Nezavist administration, demonstrate a phenomenon that mirrors the findings of Jin et al. [71]. In their study, LPS was administered to mice after lesioning the DBH‐containing (noradrenergic) neurons in the cNTS. The lesioned animals responded more avidly to LPS, with further increases in peripheral inflammatory cytokines and a reduction in the anti‐inflammatory cytokine IL‐10. Our results with Nezavist were similar, except for an increase not only in inflammatory cytokines but also an increase in IL‐10 in mice treated with LPS and Nezavist, compared to LPS alone. This difference may be due to the experimental paradigm, since in our studies, LPS was administered for 4 days, while the studies of Jin et al. [90] used a single LPS injection. We chose to examine the effects of Nezavist on LPS‐generated immune responses (cytokine levels) in a paradigm in which LPS was given daily over several days to somewhat mimic daily intake of high amounts of alcohol which would, in a repetitive manner, disrupt the integrity of the intestinal barrier to the products of the microbiome [9, 121]. The literature indicates that, compared to a single injection of LPS, three injections produce a more profound response of serum IL‐1*β*, IL‐6, TNF*α* and IL‐10 [122].

The profile of changes in cytokine levels produced by LPS and Nezavist was quite different in the CNS (hippocampus), compared to circulating levels of cytokines. In our study, the levels of IL‐6 and TNF*α* in the hippocampus were significantly increased by LPS, but no substantial additional changes occurred with Nezavist. On the other hand, administering Nezavist with LPS significantly *diminished* the LPS‐induced increase in IL‐1*β* levels in the hippocampus. In the hippocampus, as in certain other brain areas [123, 124], there are two sources of IL‐1*β*. One source is the immune cells (infiltrating macrophages/monocytes and microglia), and the other source is neurons that synthesize and release IL‐1*β* [125]. A pathway has been defined from the NTS to the hippocampus that consists of glutamatergic projections from the NTS to the Nucleus Paragigantocelluaris, followed by glutamatergic projections to the locus coeruleus, followed by noradrenergic projections to the hippocampus [126, 127]. In addition to inhibiting the activity of NE neurons in the NTS, the inhibitory GABA/somatostatin interneurons in the NTS may also affect the glutamatergic output from the NTS [114]. The downstream noradrenergic input to the hippocampus is essential for facilitating attention, cognition and behavioural changes in response to the environment [128, 129]. A significant amount of literature has linked neuronal IL‐1*β* actions in the hippocampus to both neuroprotective and neurodegenerative processes. Low levels of IL‐1*β* provide for neuronal recovery from injury and have a role resembling that of a neuromodulator [130], while high levels have been implicated in pathological processes including epilepsy, depression, memory dysfunction (Alzheimer's Disease) and Parkinsonism [131]. Although it is difficult to find reference to a direct relationship between noradrenergic neuron function in the hippocampus and IL‐1*β* synthesis or release from hippocampal neurons, such evidence has been gathered from other brain areas. Noradrenergic neurons from the A2 region *of the NTS* project to the paraventricular nucleus (PVN) of the hypothalamus and activate both oxytocin and CRF‐containing neurons. Such activation leads to the release of these hormones [120]. The noradrenergic input to the paraventricular hypothalamus also increases IL‐1*β* synthesis and release from the hypothalamic neurons [132]. It is notable that circulating CRF levels which we measured were *diminished* by treatment with LPS and further decreased by the combination of LPS and Nezavist, indicating a possible inhibition of NTS noradrenergic input to the PVN.

### 4.8 Nezavist and the Role of Vagal Activity, the NTS Noradrenergic System and Cytokines in AUD

A noradrenergic pathway also projects from the NTS to the ‘extended amygdala’ (bed nucleus of the stria terminalis, shell of the nucleus accumbens and central nucleus of the amygdala [CeA]). The noradrenergic input to the amygdala mediates local CRF signalling in the amygdala [133] and mediates behavioural responses to environmental and internal stressors [133]. The noradrenergic pathway from NTS to amygdala, together with increased CRF release in the amygdala, has been shown to be a critical component of CeA changes occurring during induction of alcohol dependence, the increased anxiety that occurs on withdrawal from alcohol, opiates and cocaine in drug‐dependent animals, and the enhanced preference for the drug on which the animal is dependent [134, 135]. Work with control and alcohol‐dependent macaques has assessed neuroadaptation, related to IL‐1*β*, in the CeA [121]. These studies found that animals who had been treated, long‐term, with alcohol, and were currently abstinent, were less sensitive to IL‐1*β* effects than control (non‐drinking) macaques when GABA release in the CeA was measured [121]. Studies on the interactions of IL‐1*β* with alcohol's effects on CeA GABAergic transmission in mice further showed that IL‐1*β* is involved in basal CeA GABAergic transmission [136]. In all, the IL‐1*β* signalling system in the brain has taken on a role as an important modulator of alcohol drinking, development of alcohol dependence and alcohol‐induced neuroimmune responses [137, 138, 139]. In addition, genetic studies in humans have identified an association between a functional polymorphism in the IL‐1*β* protein‐generating gene, and the IL‐1*β* receptor gene, with risk for AUD [140]. Particularly, the presence of a SNP (rs16944) in the sequence of the human IL‐1*β* gene increases the synthesis and release of the mature IL‐1*β* protein [141].

The literature referenced above links afferent vagal activity to modulation of noradrenergic neurons of the NTS and/or pathways from the NTS that activate noradrenergic neurons of the locus coeruleus and describes the results of inhibiting or ablating these noradrenergic projections from the brainstem to areas of the hypothalamus, amygdala and hippocampus. This information provides a mechanistic and contextual framework for explaining the effects of Nezavist to reduce alcohol dependence‐induced increases in alcohol consumption, as well as the stress coping [90, 142] actions of Nezavist as evidenced with female rats using the Porsolt test (Figure 5). Particularly important for consideration may be the participation of NTS noradrenergic activity in the cholinergic anti‐inflammatory reflex for suppressing peripheral inflammatory cytokine responses and noradrenergic (direct or indirect) projections that modulate (enhance) the production and release of IL‐1*β* in a number of brain areas [143]. The crux of our explanation of Nezavist pharmacology rests on Nezavist action in the upper intestinal tract leading to the activation of a subset of afferent vagal fibres that project to the NTS to engage local GABAergic networks and produce inhibition of the c‐Fos response to LPS, or the response to other inflammatory mediators acting on the afferent vagal projections to the cNTS. From the work of Jin et al. [71] and others [110], we hypothesize that the neurons whose c‐Fos response is diminished by Nezavist are the noradrenergic neurons (A2) of the caudal NTS regions and/or glutamatergic neurons projecting to the Nucleus Paragigantocellularis (PGi). In fact, the local injection of muscimol into the NTS was demonstrated to inhibit the activity of this NTS‐to‐PGi connection [127].

To reiterate the possible similarities of LPS action with alcohol action, one can emphasize that peripheral LPS administration to mice has been shown to produce a prolonged increase in voluntary alcohol intake and alter the electrophysiological characteristics related to alcohol reward versus aversion [138]. Gorky and Schwaber [9] have presented a summary on how gut dysbiosis generated by high levels of alcohol intake and withdrawal from alcohol consumption could engage vagal signalling. They have surmised that inflammatory events in the amygdala contribute directly to withdrawal behaviour and other signs of alcohol dependence. Although Nezavist, on its own, can activate a subset of vagal neurons, ostensibly by acting as a PAM of GABA_A_ receptors in the intestine, this stimulus does not translate into any evident c‐Fos response in the NTS. It is only in conjunction with an inflammatory signal (LPS in our studies) that the dampening effect of Nezavist occurs. If the sequelae to abstinence from alcohol, in alcohol‐dependent individuals, resemble LPS‐induced events in the NTS, then Nezavist would counter these events. The countering of inflammatory events in the CNS (i.e., suppression of IL‐1*β* production) can have a beneficial effect in both reducing withdrawal‐induced relapse to high levels of alcohol consumption, and in reducing neuronal damage caused by extended exposure to high levels of cytokines. As noted, high levels of IL‐1*β* in the CNS have been shown to negatively affect memory, responses to stressful stimuli, feeding behaviour, ‘sickness behaviour’ and so forth [130, 131]. Although there are some obvious beneficial effects of Nezavist in instances needing control of alcohol relapse behaviour, and certain aspects of neuroinflammation, there is also an obvious caveat to Nezavist use in the presence of peripheral inflammation. Although Nezavist does not produce any effect on circulating cytokine levels in non‐inflammatory conditions, it can potentiate the peripheral immune response arising from the presence of an antigenic molecule such as LPS. This phenomenon can be explained by Nezavist's interference with the function of the vagally mediated anti‐inflammatory reflex [144, 145]. It is, however, evident from our data that interference with the anti‐inflammatory reflex may be abrogated by lowering the dose of Nezavist, while the CNS anti‐inflammatory action of Nezavist may be maintained at a lower dose. Such dose–response studies, and a number of other studies substantiating relationships proposed herein to explain Nezavist actions are clearly needed. By presenting some of the results on the actions of Nezavist in this manuscript, we hope to raise the interest of other investigators (beyond ourselves) for investigating the pharmacology of Nezavist.

## 5 Summary and Speculation

In summary, we have designed and synthesized a new chemical entity (NCE), which has been characterized as a PAM at a novel site of the GABA_A_ receptors [2]. We refer to this NCE as Nezavist. Nezavist was demonstrated, in the current work, to reduce/eliminate relapse‐like increases in alcohol consumption/responding in animals made physically dependent on alcohol and tested during withdrawal. Nezavist was also shown to display actions indicative of effects on the phenomenon described as a ‘stress coping strategy’ [87, 90, 142] in female rats in the Porsolt test.

What was surprising was that the behavioural actions of Nezavist were evident in the absence of meaningful levels of Nezavist in the circulation or brain. This conundrum was resolved by demonstrating that Nezavist could activate a subset of afferent (sensory) vagal neurons innervating the upper intestinal tract. In live animals, Nezavist per se did not produce a measurable c‐Fos response in the brainstem NTS, which would indicate a vagal gut/brain signal initiated by administration of Nezavist. However, when the animal was challenged with LPS to activate a strong immune response, which involves a significant increase in vagal signalling to the NTS and increases in NTS c‐Fos expression, Nezavist was shown to significantly dampen this response. Following the burgeoning literature on the involvement of the immune system in AUD we assessed cytokine levels in the brain and in the peripheral circulation of animals treated with LPS or LPS plus Nezavist. The levels of the five cytokines measured in the brain (hippocampus) were significantly (except for IL‐18) elevated by LPS. The administration of Nezavist had a selective effect on the cytokines increased by LPS, i.e., Nezavist selectively lowered the LPS‐induced increase in hippocampal IL‐1*β*. On the other hand, in the periphery, Nezavist *potentiated* the stimulatory effect of LPS on circulating cytokines.

In Section 4, we propose how all of the measured actions of Nezavist on immune system signalling can be reconciled by postulating that Nezavist activates particular vagal afferent neurons, which then activate a sparse number of GABAergic neurons in the NTS (possibly those that also express somatostatin). These interneurons inhibit a large number of noradrenergic and/or glutamatergic neurons that are second order, output neurons from the NTS. Such a mechanism can account for both the CNS and peripheral effects of Nezavist on LPS‐generated increases in cytokine levels. In the CNS (hippocampus), the effects of Nezavist were confined to IL‐1*β*. Overexpression of IL‐1*β* in a number of brain areas has been linked to neurodegenerative disorders [131] and more recently to the effects of chronic intermittent alcohol administration in mice [146]. The alcohol administration paradigm in the studies of Patel et al. [146] resembles the paradigms used in our current work on ‘relapse’ with rats. The effect of LPS that we witnessed in the hippocampus also resembles the increases in IL‐1*β* produced by chronic alcohol administration, which were found in studies of the central amygdala and the medial prefrontal cortex of alcohol‐treated mice [146, 147]. The changes in levels of IL‐1*β* expression in the alcohol‐treated mice in those studies were noted in both microglia and neurons [136], and data were presented that the chronic alcohol treatment generated a ‘switch’ in the function of IL‐1*β* enhancing a proinflammatory phenotype [147]. Zou and Crews [148] had earlier demonstrated that alcohol induces IL‐1*β* overexpression in the hippocampus, which was linked to the suppression of neurogenesis in this brain area. The amalgam of the studies described above indicates that the induction of IL‐1*β* by alcohol may be a more general phenomenon throughout the brain, and a reduction in IL‐1*β* levels by Nezavist may be neuroprotective and ameliorative in the treatment of AUD.

Are the neuroinflammatory changes and particularly the increases in IL‐1*β* in various areas of the brain that are produced by chronic administration of alcohol, or by LPS administration, associated with alcohol craving and relapse, including the observation of increased alcohol consumption during withdrawal in an alcohol‐dependent subject? In addition to evidence presented in Section 4 [137, 138], Marshall et al. [149], in an attempt to clarify the LPS effect on alcohol drinking by mice, focused on IL‐1*β* as a possible mediator of the LPS effects. Their studies showed that chronic alcohol consumption induced an increase in IL‐1*β* levels in the amygdala, commensurate with increases in alcohol intake over successive sessions of alcohol exposure. They further showed a decrease in alcohol consumption upon bilateral amygdala injection of the IL‐1*β* receptor *antagonist*, IL‐1*β*Ra.

In all, there are a number of parallels that can be drawn between the actions of LPS and particular cytokines in the CNS during chronic alcohol consumption and on escalation of alcohol intake and relapse after a period of abstinence. Thus, our results showing Nezavist reduction of LPS‐induced elevation of IL‐1*β* levels in the brain may have a mechanistic relevance to Nezavist‐induced decreases in alcohol consumption in the models of ‘relapse’ in alcohol‐dependent rats.

The major caveat in terms of the use of Nezavist in AUD treatment emanating from our work is the finding of the potentiation, by Nezavist, of the peripheral response to administration of LPS. The increases in the levels of cytokines in the periphery over those produced by LPS alone could be expected to contribute to peripheral organ damage even though it is hard to predict whether potentiation of a lower immune challenge in the periphery (e.g., by ethanol, compared to the ip injection of LPS) would be damaging or beneficial [150]. Nezavist, in our studies, showed no effect on cytokine levels in the absence of the LPS administration. The critical question that remains is: do the Nezavist effects translate to the treatment of AUD in humans?

## Author Contributions

**Boris Tabakoff** and **Paula L. Hoffman:** conception and experimental design. **Boris Tabakoff:** manuscript writing. **Boris Tabakoff** and **Paula L. Hoffman:** manuscript editing. **Rainer Spanagel** and **Valentina Vengeliene:** studies and reports: alcohol deprivation effect. **Leandro F. Vendruscolo**, **Giordano de Guglielmo** and **Olivier George:** operant alcohol responding. **Amanda J. Roberts:** other behaviours. **Jerome D. Swinny** and **Ruolin Ma:** intestinal contractility. **Wolfgang Kunze** and **Karen‐Anne McVey Neufeld:** vagal activity. **Christina L. Lebonville** and **Howard C. Becker:** NTS activation. **Laura M. Saba:** supplemental statistical analysis. **Alexandra Dunbar:** report and figure editing.

## Funding

These studies were supported by the NIH (U44AA024905 [BT,PLH]; R44AA024905 [BT,PLH]; P50AA010761[HCB]; U54DA016511 [CLL]; U01AA014095 [HCB]; R01AA026536 [HCB]; P60AA006420 [OG, AJR]; R01AA022977[OG]; NIH Intramural Research Funding Z1A‐DA000644 [LFV]; Discovery Grant from Natural Sciences and Engineering Research Council of Canada (NSERC) [WK]).

## Ethics Statement

Animal Welfare: These studies followed national, international and/or institutional guidelines for humane animal treatment and complied with relevant legislation as indicated in Section 2 for the various studies.

## Conflicts of Interest

B.T. is the Founder and ceO of Lohocla Research Corporation; P.L.H. is the Vice President for Research of Lohocla Research Corporation. R.S. is Editor in Chief of Addiction Biology. Other authors declare no conflicts of interest.

## Supporting information


**Table S1:** Differences in alcohol consumption between animals treated with Nezavist (20 mg/kg doses) and vehicle at different alcohol concentrations.

**Figure S1:** Locomotor activity following 75‐mg/kg Nezavist in alcohol deprivation model.
**Figure S2:** Examples of immunofluorescent labelling of Iba1, CD68 and GFAP in animals of Groups A, B, C and D. Images show representative labelling with Iba1 in white, CD68 in red and GFAP in orange on sagittal sections; nuclei are labelled with DAPI and are shown in blue. Single‐channel magnifications show labelling in the hippocampus; images were taken at the position indicated by the rectangle. Note the arrows in the Iba1 channel pointing at the activated microglia.

## Data Availability

The data that support the findings of this study are available on request from the corresponding author. The data are not publicly available due to privacy or ethical restrictions.
