# Supplementary material for: Engaging Gut‐to‐Brain Signalling to Treat Alcohol Use Disorder
Source: Addict Biol. 2026 Mar 25;31(3):e70144. doi: 10.1111/adb.70144 (PMC13093268; doi:10.1111/adb.70144)
Supplement: Supplementary file 1 — Table S1: Differences in alcohol consumption between animals treated with Nezavist (20 mg/kg doses) and vehicle at different alcohol concentrations. [file ADB-31-e70144-s001.docx]

| Supplementary Table 1. Differences in Alcohol Consumption between Animals Treated with Nezavist (20 mg/kg doses) and Vehicle at Different Alcohol Concentrations | | | | | | | | |
| --- | --- | --- | --- | --- | --- | --- | --- | --- |
| timepoint | Interaction Effect Between Drug (Nezavist vs vehicle) and Alcohol Concentration (5, 10, and 20%) [p-value] | contrast | Percent Alcohol | Estimated Difference | Standard Error | DF | t-statistic | p-value |
| ADE Day 1 | 0.0507 | Nezavist - vehicle | 5 | -0.62 | 0.51 | 70.0 | -1.23 | 0.2227 |
|  |  | Nezavist - vehicle | 10 | 0.73 | 0.50 | 70.0 | 1.45 | 0.1514 |
|  |  | Nezavist - vehicle | 20 | -0.93 | 0.50 | 70.0 | -1.88 | 0.0640 |
| ADE Day 2 | 0.0107 | Nezavist - vehicle | 5 | -0.26 | 0.46 | 70.0 | -0.57 | 0.5689 |
|  |  | Nezavist - vehicle | 10 | 1.10 | 0.46 | 70.0 | 2.41 | 0.0184 |
|  |  | Nezavist - vehicle | 20 | -0.89 | 0.48 | 70.0 | -1.87 | 0.0652 |
| ADE Day 3 | 0.3633 | Nezavist - vehicle | 5 | -0.27 | 0.48 | 71.0 | -0.55 | 0.5850 |
|  |  | Nezavist - vehicle | 10 | 0.66 | 0.47 | 71.0 | 1.40 | 0.1661 |
|  |  | Nezavist - vehicle | 20 | -0.03 | 0.47 | 71.0 | -0.07 | 0.9419 |
