# Supplementary material for: Engaging Gut‐to‐Brain Signalling to Treat Alcohol Use Disorder
Source: Addict Biol. 2026 Mar 25;31(3):e70144. doi: 10.1111/adb.70144 (PMC13093268; doi:10.1111/adb.70144)
Supplement: Supplementary file 2 — Figure S1: Locomotor activity following 75‐mg/kg Nezavist in alcohol deprivation model. Figure S2: Examples of immunofluorescent labelling of Iba1, CD68 and GFAP in animals of Groups A, B, C and D. Images show representative labelling with Iba1 in white, CD68 in red and GFAP in orange on sagittal sections; nuclei are labelled with DAPI and are shown in blue. Single‐channel magnifications show labelling in the hippocampus; images were taken at the position indicated by the rectangle. Note the arrows in the Iba1 channel pointing at the activated microglia. [file ADB-31-e70144-s002.pdf]

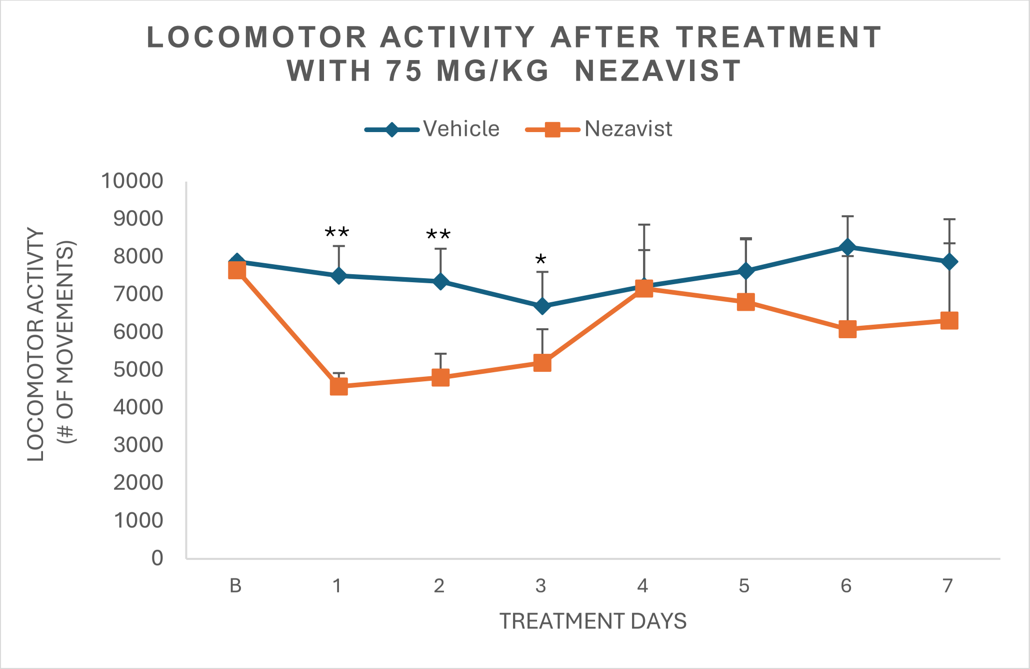

Figure 1. Locomotor Activity Following 75 mg/kg Nezavist in Alcohol Deprivation Model.

Data represent mean ± SD (n=8/group). Home cage locomotor activity was monitored with an infrared sensor connected to a recording and data storing system (Mouse-E-Motion) from 7 pm to 7 am. The device sampled every second, and the data represent number of movements. Nezavist was administered 12 hours prior to alcohol re-introduction, and at 12-hour intervals on the first and second days of alcohol re-introduction. ** P< 0.001, * P< 0.01(ANOVA and Holm-Sidak test). B = basal.

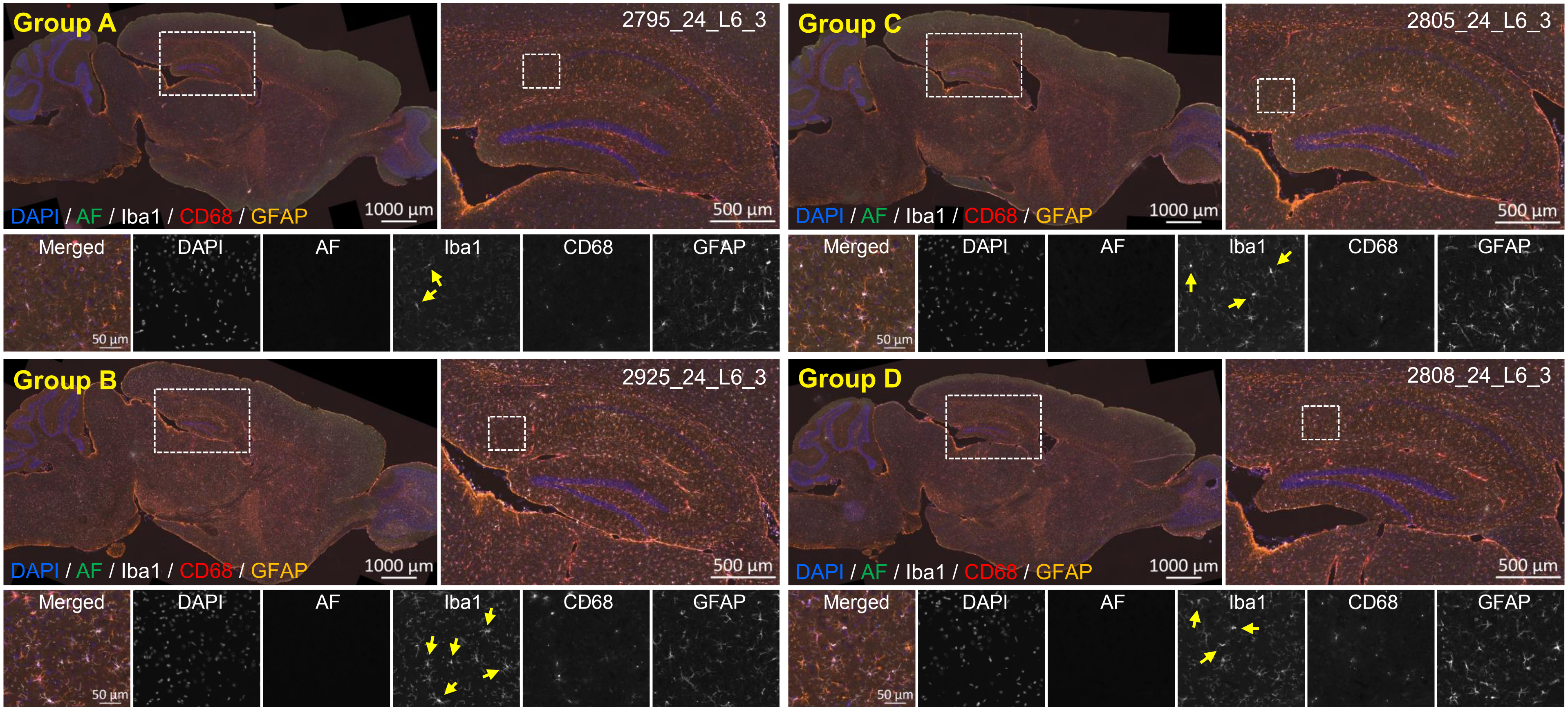

Figure 2: Examples of immunofluorescent labeling of Iba1, CD68 and GFAP in animals of groups A, B, C, and D. Images show representative labeling with Iba1 in white, CD68 in red and GFAP in orange on sagittal sections; nuclei are labeled with DAPI and are shown in blue. Single channel magnifications show labeling in the hippocampus; images were taken at the position indicated by the rectangle. Note the arrows in the Iba1 channel pointing at the activated microglia.
